# Genetic and Genomic Analyses Reveal Boundaries between Species Closely Related to *Cryptococcus* Pathogens

**DOI:** 10.1128/mBio.00764-19

**Published:** 2019-06-11

**Authors:** Andrew Ryan Passer, Marco A. Coelho, Robert Blake Billmyre, Minou Nowrousian, Moritz Mittelbach, Andrey M. Yurkov, Anna Floyd Averette, Christina A. Cuomo, Sheng Sun, Joseph Heitman

**Affiliations:** a Department of Molecular Genetics and Microbiology, Duke University Medical Center, Durham, North Carolina, USA; b Lehrstuhl für Allgemeine und Molekulare Botanik, Ruhr-Universität Bochum, Bochum, Germany; c Geobotany, Faculty of Biology and Biotechnology, Ruhr-University Bochum, Bochum, Germany; d Leibniz Institute DSMZ-German Collection of Microorganisms and Cell Cultures, Braunschweig, Germany; e Broad Institute of MIT and Harvard, Cambridge, Massachusetts, USA; Vallabhbhai Patel Chest Institute

**Keywords:** chromosomal rearrangements, fungi, genome analysis, reproductive isolation, speciation

## Abstract

Speciation is a central mechanism of biological diversification. While speciation is well studied in plants and animals, in comparison, relatively little is known about speciation in fungi. One fungal model is the *Cryptococcus* genus, which is best known for the pathogenic Cryptococcus neoformans/Cryptococcus gattii species complex that causes >200,000 new human infections annually. Elucidation of how these species evolved into important human-pathogenic species remains challenging and can be advanced by studying the most closely related nonpathogenic species, Cryptococcus amylolentus and Tsuchiyaea wingfieldii. However, these species have only four known isolates, and available data were insufficient to determine species boundaries within this group. By analyzing full-length chromosome assemblies, we reappraised the phylogenetic relationships of the four available strains, confirmed the genetic separation of C. amylolentus and T. wingfieldii (now Cryptococcus wingfieldii), and revealed an additional cryptic species, for which the name Cryptococcus floricola is proposed. The genomes of the three species are ∼6% divergent and exhibit significant chromosomal rearrangements, including inversions and a reciprocal translocation that involved intercentromeric ectopic recombination, which together likely impose significant barriers to genetic exchange. Using genetic crosses, we show that while C. wingfieldii cannot interbreed with any of the other strains, C. floricola can still undergo sexual reproduction with C. amylolentus. However, most of the resulting spores were inviable or sterile or showed reduced recombination during meiosis, indicating that intrinsic postzygotic barriers had been established. Our study and genomic data will foster additional studies addressing fungal speciation and transitions between nonpathogenic and pathogenic *Cryptococcus* lineages.

## Abstract (precis)

The evolutionary drivers of speciation are critical to our understanding of how new pathogens arise from nonpathogenic lineages and adapt to new environments. Here we focus on the Cryptococcus amylolentus species complex, a nonpathogenic fungal lineage closely related to the human-pathogenic Cryptococcus neoformans/Cryptococcus gattii complex. Using genetic and genomic analyses, we reexamined the species boundaries of four available isolates within the C. amylolentus complex and revealed three genetically isolated species. Their genomes are ∼6% divergent and exhibit chromosome rearrangements, including translocations and small-scale inversions. Although two of the species (C. amylolentus and newly described C. floricola) were still able to interbreed, the resulting hybrid progeny were usually inviable or sterile, indicating that barriers to reproduction had already been established. These results advance our understanding of speciation in fungi and highlight the power of genomics in assisting our ability to correctly identify and discriminate fungal species.

## INTRODUCTION

Speciation is the process by which two species are formed from a common ancestor and is one of the main biological processes generating biodiversity. For organisms that reproduce sexually, including most animals and plants, as well as many fungi, this process implies the development of reproductive barriers inhibiting gene flow between diverging populations. These barriers can be pre- or postzygotic, depending on whether they operate before or after fertilization. Prezygotic barriers can be environmental, including temporal and/or spatial separation of sexual reproduction among closely related species, or they can be biological, when mating is prevented through unsuccessful gamete recognition. Postzygotic reproductive barriers are often associated with the generation of hybrid progeny that are either inviable or sterile and are expected to arise as a result of divergence between nascent species. In these cases, negative epistatic interactions between mutations fixed independently in the diverging lineages are expected to play a prominent role when brought together in the same individual (known as Bateson-Dobzhansky–Müller incompatibility) (1).

For fungal species, prezygotic barriers may be achieved through, for example, loss of the pheromone and pheromone receptor interaction that usually initiates mating between cells with compatible mating types (*MAT*). With respect to postzygotic barriers, these can be achieved through several different mechanisms. First, mating type-specific transcription factors that determine compatibility after cell fusion could be incompatible between mating partners of divergent lineages, thereby preventing the formation of an active heterodimer in the zygote that is critical for sexual reproduction to proceed (reviewed in references 2 and 3). Second, the segregation of coadapted nuclear and cytoplasmic elements (e.g., mitochondria) could affect the fitness or even viability of the zygotes as well as subsequent sexual development (4–7). Third, the accumulation of genetic differences (including both sequence divergence and chromosomal rearrangements) between incipient species or diverging populations could compromise meiosis after hybridization, likely by disrupting homologous recombination and faithful chromosomal segregation, thus producing progeny with imbalanced genetic material that are either inviable or sterile (8–11). Indeed, it has been recently shown that in interspecific crosses of the yeasts Saccharomyces cerevisiae and Saccharomyces paradoxus, the majority of the observed hybrid inviability could be attributed to meiosis I chromosomal nondisjunction, the frequency of which could be significantly reduced by partially impairing the activity of genes that prevent homologous recombination between nonidentical sequences. This finding suggests that the chromosomal missegregation observed during meiosis is likely due to the presence of sequence divergence (12).

It has recently been estimated that there may be as many as 2.2 to 3.8 million fungal species in the world (13–15). Fungi can influence the recycling of nutrients in diverse ecosystems as free-living organisms (16) or can impact the health of many plants and animals positively as commensals or negatively as pathogens (17). The “definition of species” concept affects how studies defining species are carried out and therefore has a great influence on diversity studies (18). While a vast diversity in the fungal kingdom is appreciated, the definition of species and the identification of species boundaries in fungi are not always straightforward. Different approaches to defining species can result in detecting different entities. Species defined based on phenotypic or morphological variation may not necessarily be the same as species defined based on reproductive isolation (otherwise known as the biological species concept) (19). While many fungal species can be defined using the biological species concept, with robust sexual reproduction within but not between closely related species, the biological species definition frequently cannot be readily applied. For instance, it cannot be applied to define species without a known sexual cycle. Difficulties also arise because closely related fungal species may undergo hybridization and often produce viable hybrid progeny (albeit with highly reduced spore viability) that can propagate asexually through mitosis or, in rare cases, can even engage in sexual reproduction through backcrossing with the parental species (10, 11, 20, 21). An alternative approach that avoids some of these pitfalls is to use DNA sequences to differentiate populations and define species. Thus, many fungal species are now defined through phylogeny-based approaches (22), consisting of an analysis of divergence between lineages using selected DNA sequences that can distinguish a broad range of fungi (DNA barcoding [23]) or analysis performed by comparing whole-genome sequences while looking for other genomic changes, such as chromosomal rearrangements. One advantage of applying such criteria is that these genomic changes tend to occur and can be recognized before divergence has accumulated in other aspects of fungal biology, such as mating behavior or morphology. However, because slight differences can be found among the members of virtually any group of fungi, the phylogenetic species concept alone can sometimes encourage extreme division of species into ever smaller groups. Therefore, when possible, the use of a combination of approaches allows the most accurate assessment of the dynamics underlying speciation.

The Cryptococcus neoformans/Cryptococcus gattii species complex is a group of closely related basidiomycete yeasts that have been used as model organisms to study fungal pathogenesis and for antifungal drug discovery (24, 25). There are currently seven defined species within this species complex (26). They mostly infect immunocompromised hosts, with infection initiating in the lungs. If not treated, the infection disseminates to other organs, especially the brain, where it causes meningoencephalitis. Pathogenic *Cryptococcus* species collectively cause over 200,000 infections annually, placing them among the leading groups of human-pathogenic fungal species (27). While many factors that contribute to virulence have been identified in the C. neoformans/C. gattii complex, it is still not fully understood how their pathogenesis evolved. Interestingly, many of the species that are closely related to members of the pathogenic *Cryptococcus* species complex, such as Cryptococcus amylolentus, Cryptococcus depauperatus, and Cryptococcus luteus, are not known to cause disease in plants or animals (28–31) and are instead regarded as saprobes or mycoparasites (32). Among these sister species, C. amylolentus is the species most closely related to the members of the pathogenic C. neoformans/C. gattii complex. Phylogenetic analyses as well as recent extensive genomic comparison studies have shown that substantial sequence divergence and chromosomal rearrangements have accumulated since the separation of the C. neoformans/C. gattii and C. amylolentus lineages (28–31). One such rearrangement is associated with the transition from an ancestral tetrapolar breeding system (wherein two independent and unlinked mating type loci determine pre- and postfertilization compatibility [reviewed in reference 3]) to the extant bipolar breeding system of the pathogenic species that have linked mating type (*MAT*) loci (33). Such a transition of reproductive compatibility is beneficial in inbreeding mating systems (34, 35) and was likely selected for in these fungi (36). Genomic rearrangements can thus serve as direct targets for natural selection to act upon and are potentially of functional significance for virulence and the emergence of Cryptococcus neoformans/C. gattii species as major human pathogens.

A total of four isolates of the C. amylolentus species complex are available for study. These include Tsuchiyaea wingfieldii type strain CBS7118 and recently isolated strain DSM27421 (37), which, together with the two C. amylolentus strains, form the sister clade to the pathogenic *Cryptococcus* lineage (38). While isolates are routinely identified using partial ribosomal gene sequences, the type strains of C. amylolentus and T. wingfieldii were also studied using additional protein-coding genes, and the two species were considered taxonomic synonyms because of relatively low divergence among the sequences analyzed (38). However, no whole-genome sequencing and karyotypic data were available for either of the two isolates of T. wingfieldii, and it remained unclear whether they are compatible with respect to mating with other strains. Thus, the taxonomic status of strains CBS7118 and DSM27421 still needs to be fully established. Given their close phylogenic relationship with C. amylolentus, they could be closely related diverging lineages of the same species or they could represent distinct species. Furthermore, a detailed comparison of the isolates in the sister clade of the pathogenic *Cryptococcus* lineage would also allow us to identify with higher accuracy any genetic divergence and chromosomal rearrangements that have segregated within and between the pathogenic and nonpathogenic sister lineages and could thus provide further insights into the evolution of pathogenesis in the C. neoformans/C. gattii species complex.

In this study, we reexamined the species boundaries of the four isolates available within the C. amylolentus complex using phylogenetic, genetic, and genomic approaches and revealed three genetically isolated species. We show that whereas strain CBS7118 did not mate with any of the strains tested, strain DSM27421 could undergo sexual reproduction with C. amylolentus to produce spores. However, by analyzing the F1 progeny of crosses between DSM27421 and C. amylolentus strains, we show definitively the presence of postzygotic reproductive barriers between the two lineages, similar to those observed among the sister species in the C. neoformans/C. gattii species complex. These results confirm the genetic separation of C. amylolentus and strain DSM27421, for which the name Cryptococcus floricola is proposed. Additionally, we sequenced and assembled the complete genomes of isolates CBS7118 and DSM27421. Genomic comparison of the two newly assembled genomes, together with those from C. amylolentus, identified both species- and clade-specific sequence polymorphisms and chromosomal rearrangements. We discuss our findings in the context of speciation among closely related fungal lineages, including the establishment of reproductive isolation through both sequence divergence and chromosomal rearrangements.

## RESULTS

### Whole-genome phylogenetic analysis suggested three distinct species within the C. amylolentus complex.

**FIG 1:**
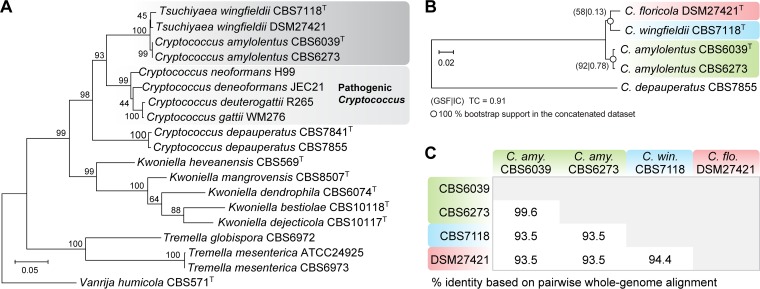
The DSM24721 strain is closely related to Cryptococcus amylolentus and C. wingfieldii. (A) Maximum likelihood (ML) phylogram inferred from a concatenated alignment of the internal transcribed spacer region (ITS1, 5.8S, and ITS2) of the *RPB1* and *TEF1* genes, showing a close phylogenetic relationship between the C. amylolentus complex and the pathogenic *Cryptococcus* species. All positions containing gaps and missing data were excluded. The tree was rooted with sequences of Vanrija humicola. There were a total of 1,164 positions in the final data set. (B) ML phylogeny reconstructed from the concatenated protein alignments of 4,896 single-copy genes shared across the studied taxa and the outgroup represented by C. depauperatus. Measures of gene support frequency (GSF) and internode certainty (IC) are shown at the nodes, and the tree certainty (TC) is given at the bottom. Branch lengths in both trees are given in number of substitutions per site. Former species names assigned to CBS7118 and DSM27421 are used in panel A, whereas the new names or new combinations proposed in this study are used in panel B. In both trees, bootstrap percentage values from 1,000 replicates are shown at the tree nodes and the type strain of each species is indicated by a superscript capital T. (C) The percentage of identical DNA base pairs is shown for each pairwise combination of the four strains.

Mittelbach et al. first isolated strain DSM27421 in 2012 from flower nectar collected in Tenerife, Canary Islands, Spain (37). This isolate was originally identified as T. wingfieldii (a taxonomic synonym of C. amylolentus) based on sequence similarity of the D1/D2 domains of the large-subunit (LSU) (26/28S) rRNA gene, which represents a barcode commonly used in yeast identification. Because the LSU nucleotide sequences of the type strains of C. amylolentus and T. wingfieldii differ only in one nucleotide and one gap, additional genes were employed to try to resolve the phylogenetic relationships of the four strains in the C. amylolentus species complex. First, a maximum likelihood (ML) gene tree was inferred using a concatenated alignment of the internal transcribed spacer region (ITS1-5.8S-ITS2) and partial sequences of the *RPB1* and *TEF1* genes (Fig. 1A). In this tree, the group consisting of T. wingfieldii DSM27421, T. wingfieldii CBS7118, C. amylolentus CBS6039, and C. amylolentus CBS6273 branched out from the other members of the *Cryptococcus* genus in a subtree that received 100% bootstrap support. However, the delimitation between T. wingfieldii and C. amylolentus remained unclear as the placement of DSM27421 and CBS7118 in a branch separate from the two C. amylolentus strains was weakly supported (Fig. 1A). The maximum pairwise distance between these four strains was 0.012 substitutions per site, which is less than, for example, the distance between the pathogenic species C. neoformans and C. deneoformans (0.028 substitutions per site).

To obtain a finer resolution of the C. amylolentus species complex, we generated Illumina paired-end sequencing data and draft genome assemblies for T. wingfieldii strains CBS7118 and DSM27421. A maximum likelihood phylogeny inferred from the alignment of 4,896 single-copy genes shared among the studied taxa and an outgroup species, C. depauperatus (CBS7855), revealed two robustly supported branches: one containing C. amylolentus strains CBS6039 and CBS6273 and the other containing the two T. wingfieldii strains DSM27421 and CBS7118 (Fig. 1B). Because bootstrap values from concatenated data sets can be misleading, we also bootstrapped well-supported single-copy gene trees (i.e., with >50% of 1,000 bootstrap replicates at all nodes) and used them to infer both the gene support frequency (GSF) that indicates the percentage of individual gene trees that contain a given bipartition and the internode certainty (IC) that quantifies the certainty of a bipartition (39, 40). In this analysis, 92% of the well-supported gene trees recovered the C. amylolentus clade, but the clade containing T. wingfieldii DSM27421 and CBS7118 strains was recovered in only 58% of the supported trees. Therefore, while the gene trees for the two C. amylolentus isolates showed strong support for a single clade, as expected for a single species, the evidence supporting a single clade for the two T. wingfieldii strains was not as strong, raising the possibility that they may represent separate species. In line with this, while the two C. amylolentus isolates shared 99.6% sequence identity at the whole-genome level, the CBS7118 and DSM27421 isolates were clearly more divergent from each other, sharing only 94.4% gene identity, with each only sharing 93.5% identity with C. amylolentus (Fig. 1C). Together, our data suggest that there are three phylogenetically distinct species present in this complex, with CBS6039 and CBS6273 representing C. amylolentus, CBS7118 representing the formerly described T. wingfieldii that we renamed the new taxonomic combination Cryptococcus wingfieldii, and DSM27421 representing a third, as-yet-undescribed species that we named Cryptococcus floricola.

However, such sequence divergence does not necessarily prove that these isolates represent fully established, reproductively isolated species. Thus, we further analyzed several aspects of these strains, including (i) their ability to undergo interspecies hybridization and, when hybridization did occur, the effects of meiosis on the viability and genetic composition of any hybrid progeny; (ii) the presence of chromosomal rearrangements and, together with sequence divergence, their collective effects on meiosis and chromosomal segregation; and (iii) whether their phenotypic characteristics and physiological profiles are consistent with their belonging to distinct species.

### Postzygotic but not prezygotic reproductive barriers have been established between DSM27421 and C. amylolentus.

**FIG 2:**
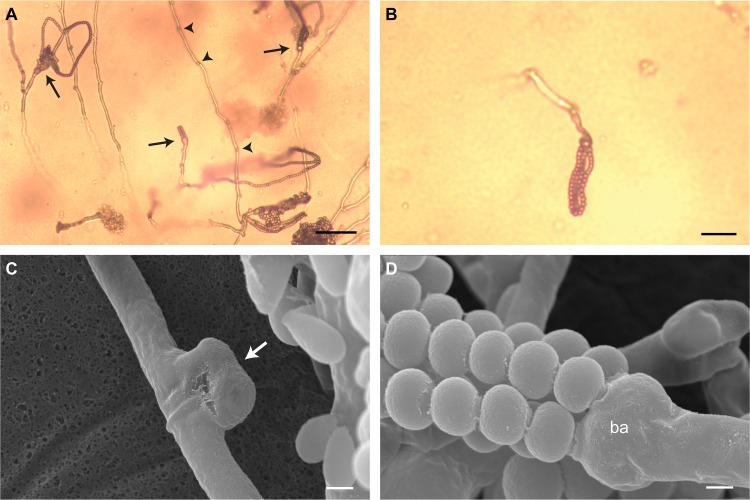
Sexual structures formed in a cross between C. amylolentus CBS6039 and DSM27421. (A) Light micrograph of the hyphae and basidia with spore chains (arrows) that formed during this cross on V8 (pH = 5) mating medium. Note the presence of clamp connections (arrowheads) at the junction of the hyphal cells. (B) Light micrograph of a basidium and spore chains at a higher magnification. (C and D) Scanning electron micrographs at a magnification of ×10,000 showing details of a fused clamp cell (white arrow) (C) and four chains of spores arising from the apical surface of a basidium (ba) (D). Scale bars represent 50 μm in panel A, 20 μm in panel B, and 1 μm in panels C and D.

In basidiomycete yeasts, mating compatibility is determined at two different levels. First, cells must undergo reciprocal exchange of mating pheromones that are recognized by specific receptors, both encoded by the pheromone/receptor (*P*/*R*) locus. After cell fusion, distinct homeodomain transcription factors, encoded by the *HD* locus of each mating partner, interact to initiate sexual development. For successful mating and completion of the sexual cycle, both compatibility factors must be heterozygous in the product of mating (e.g., zygote or dikaryon) (3). Therefore, prezygotic barriers are absent when a cross between two strains results in normal development of sexual structures. For *Cryptococcus*, complete sexual development includes hyphae with complete clamp connections, basidia, and basidiospores. To assess this, CBS7118 and DSM27421 were crossed with each other and with all of the available strains of C. amylolentus (i.e., the two parental isolates, CBS6039 and CBS6273, and both F1 and F2 progeny derived from a cross between these two strains; Table 1). All combinations of A1 or A2 (*P*/*R* locus) and B1 or B2 (*HD* locus) *MAT* alleles were accounted for in the C. amylolentus strains employed for crosses with CBS7118 and DSM27421. Following incubation for 2 weeks at room temperature in the dark, no mating was observed in any of the crosses that involved CBS7118; hence, this strain is either sterile or incompatible with all of the strains tested (Table 1). On the other hand, strain DSM27421 successfully mated with the C. amylolentus tester strains with the A1B1 or A1B2 mating type but not those with the A2B1 or A2B2 mating type, indicating that this strain has an A2B3 mating genotype. Sparse aerial hyphae were observed that were present around the periphery of the mating colonies and projected away from the agar surface. The aerial hyphae displayed prominent fused clamp connections indicative of dikaryon formation (Fig. 2A and C), and the distal ends of the hyphae differentiated into round, unicellular basidia (Fig. 2B and D). Four long chains of spores budded from the apical surface of the basidium (Fig. 2D), with the four most distal spores remaining firmly attached to each other. The sexual structures observed in heterospecific crosses (i.e., crosses between strains of different species, e.g., DSM27421 × CBS6039) were morphologically indistinguishable from those previously reported for the C. amylolentus CBS6039 × CBS6273 conspecific cross (29) but were visually much less abundant.

**TABLE 1:** Strains used in this study

Strain^*a*^	Organism/species	Mating type	Source of strain
CBS7118	*Cryptococcus wingfieldii*		Frass from scolytid beetles collected in South Africa
DSM27421	*Cryptococcus floricola*	A2B3	Nectar from the flower *Echium leucophaeum* collected in Tenerife, Spain
CBS6039	*Cryptococcus amylolentus*	A1B1	Frass of the beetle *Enneadesmus forficulus* collected in South Africa
CBS6273	*Cryptococcus amylolentus*	A2B2	Frass of the beetle *Sinoxylon ruficorne* collected in South Africa
**SSA790**	*Cryptococcus amylolentus*	A1B2	Progeny of CBS6039 × CBS6273
**SSB821**	*Cryptococcus amylolentus*	A2B1	Progeny of CBS6039 × CBS6273
**SSC103**	*Cryptococcus amylolentus*	A1B1	Progeny of SSA790 × SSB821
SSC104	*Cryptococcus amylolentus*	A2B2	Progeny of SSA790 × SSB821
SSC118	*Cryptococcus amylolentus*	A2B2	Progeny of SSA790 × SSB821
SSC119	*Cryptococcus amylolentus*	A2B2	Progeny of SSA790 × SSB821
**SSC120**	*Cryptococcus amylolentus*	A1B2	Progeny of SSA790 × SSB821
SSC123	*Cryptococcus amylolentus*	A2B1	Progeny of SSA790 × SSB821
SSC124	*Cryptococcus amylolentus*	A2B2	Progeny of SSA790 × SSB821
**SSC125**	*Cryptococcus amylolentus*	A1B1	Progeny of SSA790 × SSB821
SSC128	*Cryptococcus amylolentus*	A2B1	Progeny of SSA790 × SSB821
**SSC129**	*Cryptococcus amylolentus*	A1B1	Progeny of SSA790 × SSB821
**SSC130**	*Cryptococcus amylolentus*	A1B2	Progeny of SSA790 × SSB821

a Strains selected for Illumina whole-genome sequencing are indicated in boldface.

For full interfertility, spore formation and viability should occur at similar rates in conspecific and heterospecific crosses. Diminished spore formation or viability in a heterospecific cross is indicative of a postzygotic barrier. It was shown previously that the conspecific cross between C. amylolentus CBS6039 and CBS6273 showed an average spore germination rate of 50%, increasing to 64% in spores dissected from F1 intercrosses (33). In contrast, heterospecific crosses between DSM27421 and C. amylolentus showed an overall germination rate of only 10% (*n* = 846 spores from 28 basidia of 6 independent crosses). The average germination rate per basidium for each cross ranged from <1% to 22% (Table 2). Interestingly, spores from the cross between DSM27421 and CBS6039 had the lowest germination rate, with only one spore germinating among 153 spores dissected from 6 basidia. At the other end of the spectrum, the cross between DSM27421 and C. amylolentus progeny SSC125 produced 3 basidia with a germination rate of at least 20%, although a fourth basidium from this cross yielded no viable spores (Table 2). Taking the results together, the lower number of spores and the high percentage of inviable progeny indicate that C. amylolentus and DSM27421 exhibited considerable intrinsic postzygotic isolation, further supporting the assignment of DSM27421 to a different biological species.

**TABLE 2:** Germination rates of spores dissected from crosses between DSM27421 and *C. amylolentus*

Cross	Basidium no.	No. of spores plated	No. (%) of spores germinated^*a*^	Avg germination rate (%)/basidium
DSM27421 (A2B3) × CBS6039 (A1B1)	1	11	0	0.57
	2	16	0	
	3	42	0	
	4	29	1 (3)	
	5	28	0	
	6	27	0	

DSM27421 (A2B3) × SSC103 (A1B1)	1	10	0	5.79
	2	48	0	
	3	17	1 (6)	
	4	46	0	
	5	39	9 (23)	

DSM27421 (A2B3) × SSC120 (A1B2)	1	14	0	16.50
	2	34	7* (21)	
	3	63	29* (46)	
	4	36	0	
	5	34	11 (32)	
	6	22	0	

DSM27421 (A2B3) × SSC125 (A1B1)	1	9	0	22.17
	2	56	20 (36)	
	3	56	11 (20)	
	4	15	5 (33)	

DSM27421 (A2B3) × SSC129 (A1B1)	1	19	3* (16)	3.95
	2	56	0	
	3	29	0	
	4	53	0	

DSM27421 (A2B3) × SSC130 (A1B2)	1	10	0	12.82
	2	14	0	
	3	13	5 (38)	

a Progeny selected for Illumina whole-genome sequencing are indicated with asterisks.

### Comparison of chromosome-level genome assemblies revealed significant differences between the three species.

Postzygotic reproductive isolation between C. amylolentus and DSM27421 could be attributed to genetic incompatibilities. For example, large chromosomal rearrangements could impose a significant barrier through the generation of unbalanced progeny. To investigate this, chromosome-level assemblies for DSM27421 and CBS7118 were generated using Pacific Biosciences (PacBio) and Oxford Nanopore Technologies (ONT) long-read sequencing platforms and were subsequently compared to the available C. amylolentus CBS6039 reference assembly (GenBank accession no. GCA_001720205). For each strain, we obtained more than 150× read depth from various PacBio and ONT sequencing runs and used different assembly strategies with Canu to account for the various read lengths and error rates of the generated data (see Table S1 at https://figshare.com/s/80a83fe2c088854e7dee). Among the resulting assemblies, those with a lower number of contigs and higher base-level accuracy were selected for further analysis after multiple iterations of Illumina-read-based error correction using Pilon (see Materials and Methods for details).

**FIG 3:**
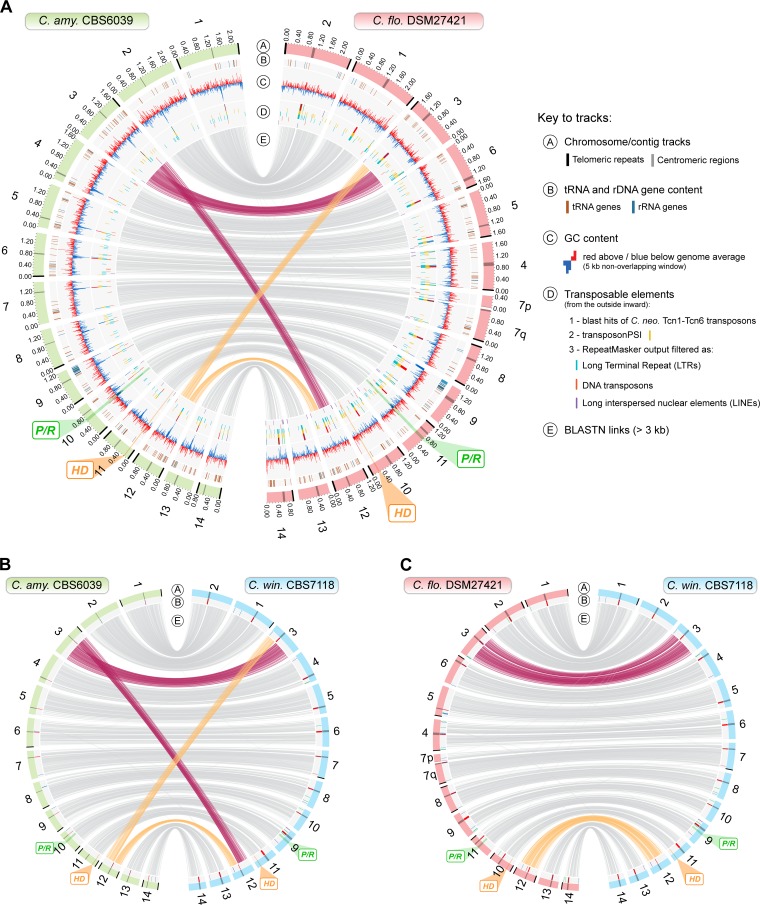
Genome-wide comparison between C. amylolentus, DSM24721, and CBS7118. A Circos plot comparing CBS6039 and DSM27421 genome assemblies is shown. Chromosome 7 of DSM27421 is broken into two contigs (7p and 7q), with centromere-specific transposable elements identified at one end of each contig. Of note are a high transposable element load in the DSM27421 genome compared to CBS6039 (track D) and a reciprocal translocation between chromosomes 3 and 12 with the breakpoint mapping within the centromere. (B and C) This translocation is also shared between CBS6039 and CBS7118 (B), whereas the DSM27421 and CBS7118 genomes are syntenic overall as shown by the links representing collinearity of genomic regions (track E) (C). Small inversions are not represented (see Fig. S4). The chromosomal locations of the *P*/*R* and *HD* mating type loci are highlighted in green and orange, respectively. Other genomic features, such as tRNA and rRNA gene content, GC content, and transposable elements, are depicted in different tracks as given in the key. Expanded views of panels B and C are presented in Fig. S3.

The final genome assemblies of DSM27421 and CBS7118 were approximately 21.7 and 20.8 Mb in size and consisted of 15 and 14 nuclear contigs, respectively, plus the mitochondrial genome (see Table S1 at https://figshare.com/s/80a83fe2c088854e7dee). Fluorescence-activated cell sorter (FACS) analyses were consistent with the two strains being haploid (Fig. S1), and contour-clamped homogeneous electric field (CHEF) electrophoresis confirmed that DSM27421 and CBS7118 have 14 chromosomes each, as does C. amylolentus (Fig. S2) (33). This indicates that all but one of the chromosomes in both strains were assembled into single contigs. Indeed, only chromosome 7 of DSM27421 was found to be fragmented into two contigs (7q and 7p; Fig. 3A and Fig. S2), with both representing arms of the same chromosome broken at the centromere. This was confirmed from chromoblot hybridization analyses using probes specific to the opposite ends of each contig (Fig. S2D and S2E) and by *in-silico* detection of regions highly enriched with long terminal repeat (LTR) retrotransposons (Fig. 3) at one end of each of the contigs. Such LTR-rich regions in C. amylolentus and C. neoformans were previously shown to be associated with centromeric regions (33, 41, 42). Finally, we annotated protein-coding genes and tRNA and rRNA genes, identified transposable elements (TE) and telomeric repeats, and predicted centromeric regions (see Materials and Methods and Fig. 3) (see also Fig. S3 as well as Tables S1, S2, and S3 at https://figshare.com/s/80a83fe2c088854e7dee).

10.1128/mBio.00764-19.1 FIG S1 Ploidy determination by FACS for strains used in this study. C. deneoformans JEC21 (Dα) and C. deneoformans XL143 (αDDα) were used as haploid and diploid controls, respectively. Approximately 10,000 cells were analyzed. The propidium iodide area (PI-A) is shown on the *x*-axis. Download FIG S1, PDF file, 0.6 MB. Copyright © 2019 Passer et al. 2019 Passer et al. This content is distributed under the terms of the Creative Commons Attribution 4.0 International license.

10.1128/mBio.00764-19.2 FIG S2 Validation of the DSM27421 genome assembly by chromoblot analysis. (A) Electrophoretic karyotypes (CHEF) of C. amylolentus (CBS6039 and CBS6273), DSM27421, and CBS7118 and selected progeny derived from a conspecific cross (SS120) and a heterospecific cross (ARP60). Chromosomes of Saccharomyces cerevisiae served as size markers. (B and C) The sizes and numbers of the bands on the CHEF gel for DSM27421 and CBS7118, respectively, are in overall agreement with the contig size obtained from genome sequencing, except for the chromosomes/contigs containing the ribosomal DNA (rDNA) array and chromosome 7 of DSM27421, which is broken into two contigs (7q and 7p; an asterisk indicates that the size is underestimated). (D) Circos plot showing the genome of DSM27421 assembled into 15 contigs (track A) and the position of Southern hybridization probes used for the chromoblot analysis (shown as bars in track B and color coded in correspondence with their respective contigs). Two probes targeting different arms of the same chromosome were used for each contig. The black vertical bars at contig ends indicate the presence of telomeric repeats, and gray bars indicate the positions of the predicted centromeres. (E) Results of Southern hybridization using the probes described in the panel D legend. In each row, the image on the far left is an ethidium bromide (EtBr) image of the CHEF gel used for the chromoblot analysis, with DSM27421 on the right and S. cerevisiae on the left serving as a size ladder. Probes that hybridized to the same chromosome are grouped together. The labels at the bottom of the hybridization images indicate the names of the probes as described in the panel D legend. It should be noted that the probes targeting chromosome 9 (9a and 9b) did not work in our chromoblot analyses. That could have been due to the fact that chromosome 9 contains the rDNA array and the fact that because of array expansion and contraction, the population used to make CHEF plugs was composed of cells with various numbers of repeats in the array and consequently had variants of chromosome 9 of various sizes that do not form a sharp band in the CHEF analysis. Download FIG S2, PDF file, 0.1 MB. Copyright © 2019 Passer et al. 2019 Passer et al. This content is distributed under the terms of the Creative Commons Attribution 4.0 International license.

10.1128/mBio.00764-19.3 FIG S3 Genomic features of C. amylolentus, DSM2741, and CBS7118 assemblies and overall synteny comparison. (A and B) Extended views of the images shown in Fig. 3B and C, showing genomic features and the overall genome synteny of CBS6039 versus CBS7118 and DSM27421 versus CBS7118, respectively. (C and D) Linear plots showing the chromosomal position of centromeres, the rDNA array, and *MAT* loci in DSM27421 (C. floricola sp. nov.) (C) and CBS7118 (C. wingfieldii comb. nov.) (D). Chromosomes are color coded based on their synteny with C. amylolentus CBS6039 chromosomes, except in panel E, where DSM27421 was used as reference. A reciprocal translocation between chromosomes 3 and 12 seems to have been driven by intercentromeric recombination and differentiates C. amylolentus from the other two sibling species. For simplicity, small inversions are not represented (see Fig. S4). Download FIG S3, PDF file, 1.9 MB. Copyright © 2019 Passer et al. 2019 Passer et al. This content is distributed under the terms of the Creative Commons Attribution 4.0 International license.

Although they were found to share the same number of chromosomes, whole-genome comparisons revealed several structural differences between C. amylolentus and the two other strains, CBS7118 and DSM27421. These differences included the following: (i) several small-scale intrachromosomal inversions mostly occurring at subtelomeric regions (Fig. S4) and at the *P*/*R MAT* locus (see below); (ii) a higher number of TE (see Table S2 at https://figshare.com/s/80a83fe2c088854e7dee) mainly associated with longer centromeric regions in DSM27421 and CBS7118 than in C. amylolentus (Fig. S5; see also Table S3 at https://figshare.com/s/80a83fe2c088854e7dee); and importantly, (iii) a reciprocal translocation involving chromosomes 3 and 12 that distinguishes C. amylolentus from the other two strains. Interestingly, the breakpoint of the translocation is located within the centromeres, suggesting that the translocation likely resulted from intercentromeric ectopic recombination mediated by common transposable elements present within the centromeres (Fig. 3; see also Fig. S3).

10.1128/mBio.00764-19.4 FIG S4 Synteny analysis highlighting diverged and inverted genomic regions between C. amylolentus, DSM2741, and CBS7118. Collinear (light blue) and inverted (red) genomic regions are shown along the length of each chromosome for each of the strains (in the same order in all chromosomes). Chromosome/contig numbers in each species are indicated at the top, with an added ‘r’ indicating contigs with reversed orientation, and are color coded as described for Fig. S2C. Note the presence of a higher number of inversions and divergent regions (white spaces interleaved within blocks of collinearity) at subtelomeric regions, predicted centromeres, and the *P*/*R MAT* locus. Other features are given in the key. Download FIG S4, PDF file, 2.9 MB. Copyright © 2019 Passer et al. 2019 Passer et al. This content is distributed under the terms of the Creative Commons Attribution 4.0 International license.

10.1128/mBio.00764-19.5 FIG S5 Centromere length and synteny comparison among species. (A) A synteny comparison across the centromeric region of C. amylolentus chromosome 1 is given as an example to illustrate the distinctive differences in the lengths of C. amylolentus centromeres compared to those of DSM27421 and CBS7118. The centromeric regions are depicted as yellow boxes showing their respective lengths defined as the distance between conserved *CEN-*flanking ORFs (left, dark green; right, dark red) (see Table S3 for additional details). Vertical bars connect regions of similarity (BLASTN hits of >1 kb) with the same (blue) or inverted (red) orientation. (B) Comparison of the predicted centromere lengths among the indicated species. Each dot represents one centromere (those depicted in panel A are shown as yellow dots), and the horizontal red lines depict the mean centromere lengths of the corresponding species. Only 13 predicted centromeres are plotted for C. floricola DSM27421 because *CEN7* is incomplete. Other *Cryptococcus* species used in this comparison are Cryptococcus deuterogattii (*C. deu.*), C. neoformans (*C. neo.*). and C. deneoformans *(C. den.*), with *CEN* length data obtained from reference 42. Different capital letters at the top indicate significantly different means (Tukey-Kramer HSD test, *P < *0.05). Download FIG S5, PDF file, 0.7 MB. Copyright © 2019 Passer et al. 2019 Passer et al. This content is distributed under the terms of the Creative Commons Attribution 4.0 International license.

**FIG 4:**
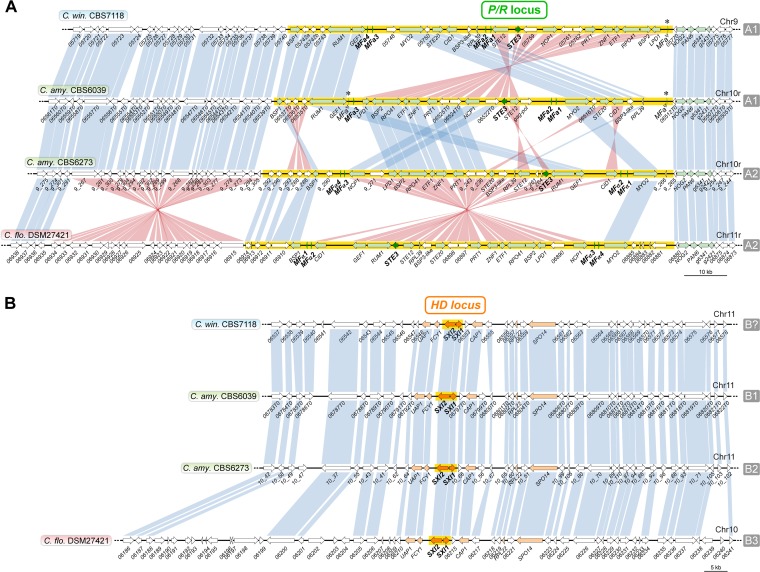
Comparison of the *MAT* loci among the studied *Cryptococcus* strains. (A and B) Synteny maps of the *P*/*R* (A) and *HD* (B) loci in C. amylolentus (CBS6039 and CBS6273), CBS7118, and DSM27421. Mating pheromone/receptor (*MFA*/*STE3*) genes and *HD* genes are depicted by dark green and dark orange arrows, respectively, showing the direction of transcription. Additional genes that are present in the fused *MAT* locus in the pathogenic *Cryptococcus* species are shown in light orange or green, while others are shown in white. Vertical blue bars connect orthologs with the same orientation, while pink bars indicate inversions. The regions spanning the proposed *HD* and *P*/*R* loci are highlighted in yellow. The structure of the *HD* locus is largely conserved among the four species, but the *P*/*R* locus underwent several gene rearrangements, even between strains of the same *P*/*R* mating-type (see Fig. S7 for details). Pheromone gene remnants (*MF*a^ψ^) in CBS7118 and CBS6039 strains are indicated by asterisks.

Among the four strains analyzed, only CBS7118 failed to demonstrate sexual reproduction. We therefore asked whether this strain retained intact *MAT* loci or if it had lost some *MAT* genes as part of gross deletions or chromosomal rearrangements. It was shown previously that the *P*/*R* and *HD MAT* loci in C. amylolentus are located on different chromosomes (33). BLAST searches using the C. amylolentus pheromone receptor (*STE3*) and *HD1*/*HD2* genes as query confirmed that the same holds true in both DSM27421 and CBS7118—the *P*/*R* locus is located on chromosomes 11 and 9 and the *HD* locus on chromosomes 10 and 11, respectively (Fig. 3; see also Fig. S3 and S4). In addition to other genes, the *P*/*R* locus of each strain contains a single mating pheromone receptor gene (*STE3*) and three to four identical putative pheromone precursors genes with a C-terminal CAAX domain such as is characteristic of fungal mating pheromones (3, 43), all apparently intact (Fig. 4A; see also Fig. S6). Further comparison of pheromone precursor proteins found that CBS7118 and C. amylolentus CBS6039 shared identical proteins, as did DSM27421 and C. amylolentus CBS6273 (Fig. S6B). Similarly, phylogenetic analyses revealed that the *STE3* alleles from C. amylolentus CBS6039 and CBS7118 group with A2/**a** alleles from more distantly related species, whereas the *STE3* alleles of C. amylolentus CBS6273 and DSM27421 clustered with other A1/α alleles (Fig. S6A). Such trans-specific polymorphism is typical of basidiomycete pheromone receptor genes and is expected for genes ancestrally recruited to the *MAT* locus and maintained across speciation by balancing selection (3, 36, 44–47).

10.1128/mBio.00764-19.6 FIG S6 Phylogeny of the pheromone receptor (*STE3*) and predicted pheromone precursor genes. (A) Phylogeny based on the nucleotide sequences of *STE3*α (A1) and *STE3*a (A2) genes from several Tremellales representatives. The *STE3* alleles of C. amylolentus, C. wingfieldii (CBS7118), and C. floricola (DSM27421) are associated with strains of the opposite mating types in other species as a result of the original arbitrary assignment of mating types in C. amylolentus based on mating tests (indicated by asterisks; e.g. the *STE3*α/A1 is found in the A2 strains CBS6273 and DSM27421). (B) Sequence alignment and comparison of MFα and MFa pheromone precursors from C. neoformans, C. amylolentus, DSM27421, and CBS7118. A consensus sequence is given at the top, and the black arrow denotes the predicted cleavage site giving rise to the peptide moiety of the mature pheromone. Download FIG S6, PDF file, 0.6 MB. Copyright © 2019 Passer et al. 2019 Passer et al. This content is distributed under the terms of the Creative Commons Attribution 4.0 International license.

10.1128/mBio.00764-19.7 FIG S7 Putative inversion events leading to the extant *P*/*R* locus configuration in C. wingfieldii CBS7118 and C. floricola DSM27421. (A) Compared to C. amylolentus CBS6039, the extant *P*/*R* locus configuration in C. wingfieldii CBS7118 appears to result from two successive inversions spanning the chromosomal segments highlighted in orange. (B) The *P*/*R* locus of C. floricola DSM27421 differs from that of C. amylolentus CBS6273 by a single large inversion spanning the chromosomal region highlighted in orange. In both cases, the breakpoints of these inversions were found to be associated with the presence of identical pheromone genes that may constitute large inverted repeats that facilitated inversion. Download FIG S7, PDF file, 1.2 MB. Copyright © 2019 Passer et al. 2019 Passer et al. This content is distributed under the terms of the Creative Commons Attribution 4.0 International license.

In basidiomycete species, the *P*/*R* locus usually exhibits synteny breaks and sequence divergence between opposite mating types, while synteny is often more conserved across species when *P*/*R* regions of the same mating type are compared (48–51). This is presumably associated with suppression of recombination between mating types at the *P*/*R* locus. Accordingly, while extensive gene shuffling has occurred between the *P*/*R* loci of C. amylolentus CBS6039 and CBS6273 (Fig. 4A), the extant rearrangements between CBS6039 and CBS7118 can be simply explained by two successive inversions in CBS7118, the first spanning the chromosomal segment between the *MYO2*/*05748* and *RPL39* genes and the second involving the region between the *MYO2*/*05748* and *LPD1* genes (Fig. S7A). The rearrangement of the *P*/*R* locus between CBS6273 and DSM27421 is even simpler. There is a single large inversion that spans the region from the left pair of pheromone genes all the way to the right pair of pheromone genes (Fig. 4A; see also Fig. S7B). Eighteen genes are predicted within this inverted region in DSM27421, all of which have corresponding orthologs in CBS6273. In contrast, two genes of unknown function in CBS6273 that lie between *STE3* and *STE12* are absent in the other three strains. Interestingly, the breakpoints of these inversions seem to be associated with the presence of identical and divergently oriented pheromone genes (or their remnants) (Fig. 4A). It is therefore likely that these genes acted as inverted repeats mediating the formation of intrachromosomal inversion loops, thereby facilitating the close apposition of the respective regions and enabling recombination. We defined the boundaries of the *P*/*R* locus in DSM27421 and CBS7118 with respect to the same regions previously assigned in C. amylolentus (highlighted with a yellow background in Fig. 4A) (33). It should be noted, however, that the *P*/*R* locus in DSM27421 may extend further to the left flank given the presence of yet another inversion specific to this species. The subsequent characterization of these inversion breakpoints at the nucleotide level revealed two genes (*06914* and *06935*) that shared 98% sequence identity and were inverted relative to one another (Fig. 4A), possibly facilitating this rearrangement.

The *HD* locus is smaller than the *P*/*R* locus, and its gene content is largely conserved among the four strains, with divergence primarily occurring at the nucleotide level (Fig. 4B). An exception is the presence of one predicted gene that lies upstream of *CAP1* in the two C. amylolentus strains, which is absent in CBS7118. Although this additional gene is also missing from DSM27421, this strain also has extensive sequence added to the left of the genes *06787* and *06789* (CBS6039 nomenclature). While this could indicate an expansion of the *HD MAT* locus in DSM27421, determining its precise length will require the analysis of additional isolates as they become available. Therefore, considering the present data, the *HD* locus most likely includes only the *SXI1* (*HD1*) and *SXI2* (*HD2*) genes or encompasses a few more genes on either side of the *SXI1*/*SXI2* gene core, as previously proposed for C. amylolentus (33) (Fig. 4B). Among these, the Sxi1 and Sxi2 protein sequences differed considerably among the four strains, consistent with their playing critical roles in mating type determination. For instance, the Sxi1 protein of C. amylolentus CBS6039 shows only 91.8%, 88.7%, and 86.5% sequence similarity with the Sxi1 proteins of CBS6273, CBS7118, and DSM27421, respectively.

Taking the results together, we obtained complete genome assemblies for DSM27421 and CBS7118 and found substantial genomic differences between these two strains and C. amylolentus, which is consistent with the hypothesis that DSM27421 and CBS7118 are distinct species. In addition, we found no clear evidence suggesting that CBS7118 was sterile as a result of mutations in the mating type-determining genes.

### Genotypic and phenotypic analysis of the progeny from the crosses between C. amylolentus and C. floricola DSM27421.

**FIG 5:**
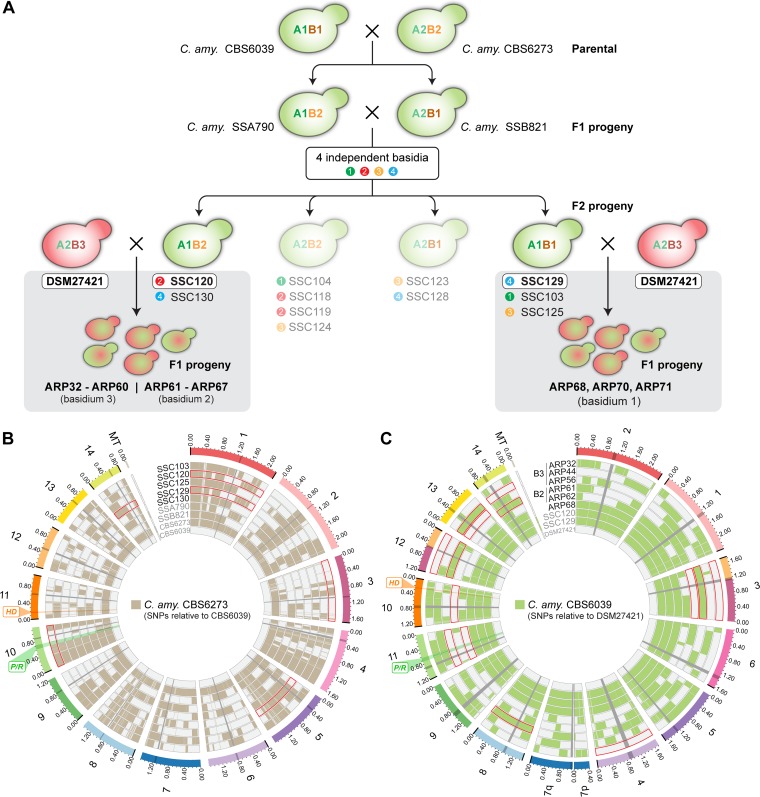
Hybrid progeny show lower levels of recombination. (A) The ancestry of the progeny set examined in this study is depicted. The C. amylolentus F2 progeny analyzed are derived from four independent basidia, and four different mating types were scored among the members of this progeny set. Of these, SSC120 and SSC129 were crossed with DSM27421. Basidium 3 from the SSC120 × DSM27421 cross produced progeny ARP32 to ARP60; basidium 2 from that same cross produced progeny ARP61 to ARP67. Basidium 1 from the SSC129 × DSM27421 cross produced progeny ARP68, ARP70, and ARP71. Illumina data were obtained for all strains except those that could not cross with DSM27421 (displayed with faded colors). (B and C) Recombination plots of the meiotic progeny derived from C. amylolentus conspecific crosses (B) and from heterospecific crosses of DSM27421 with C. amylolentus (C). SNP distributions along the length of each chromosome for each strain are colored as shown in the key. Regions of haplotype change are indicative of recombination. No crossovers were detected for several chromosomes (tracks enclosed by red lines), particularly in the heterospecific cross. For simplicity, additional basidiospores with identical genotypes (see Table 3) were not plotted in the figure.

Samples of progeny derived from two heterospecific crosses of DSM27421 and C. amylolentus were characterized further. While many crosses had low rates of spore production, we specifically collected progeny from basidia 2 and 3 of the DSM27421 × SSC120 cross that had relatively high germination rates and from basidium 1 of the DSM27421 × SSC129 cross (Fig. 5A) (Tables 2 and 3). In all, 39 progeny were collected for analysis. The *TEF1* gene, encoding translation elongation factor 1α, was initially used as a marker in a PCR-restriction fragment length polymorphism (PCR-RFLP) assay to identify different genotypes among the progeny of the same basidium (Table 3). The progeny from basidium 2 of the DSM27421 × SSC120 cross had inherited *TEF1* from only DSM27421, and the progeny from basidium 1 of the DSM27421 × SSC129 cross had inherited *TEF1* from only SSC129. However, a mix of parental *TEF1* alleles were found in the progeny from basidium 3 of the DSM27421 × SSC120 cross, indicating that at least two genotypes were represented in this set.

**TABLE 3:** Mating ability and genotypic characterization of progeny from crosses between *C. amylolentus* and DSM27421

Origin	Strain^*a*^	Mating phenotype^*b*^	Mating genotype	*TEF1* genotype	mitSSU genotype
SSA790 (A1B2) × SSB821 (A2B1)	SSC120	A1B2	A1B2	0	0
SSA790 (A1B2) × SSB821 (A2B1)	SSC129	A1B1	A1B1	0	0
Parental	DSM27421	A2B3	A2B3	1	1
DSM27421 (A2B3) × SSC120 (A1B2), basidium 3	**ARP32**		A1B2	0	1
	ARP33	A1B3	A1B3	1	1
	**ARP34**		A1B2	0	1
	**ARP35**		A1B2	0	1
	**ARP36**		A1B2	0	1
	ARP37	A1B3	A1B3	1	1
	**ARP38**		A1B2	0	1
	ARP39		A1B2	0	1
	ARP40	A1B3	A1B3	1	1
	ARP41		A1B2	0	1
	ARP42		A1B2	0	1
	ARP43		A1B2	0	1
	**ARP44**	A1B3	A1B3	1	1
	**ARP45**	A1B3	A1B3	1	1
	ARP46		A1B2	0	1
	ARP47	A1B3	A1B3	1	1
	ARP48	A1B3	A1B3	1	1
	ARP49	A1B3	A1B3	1	1
	ARP50		A1B2	0	1
	ARP51	A1B3	A1B3	1	1
	ARP52	A1B3	A1B3	1	1
	ARP53		A1B2	0	1
	ARP54	A1B3	A1B3	1	1
	ARP55	A1B3	A1B3	1	1
	**ARP56**	A2B2	A2B2	0	1
	ARP57	A1B3	A1B3	1	1
	ARP58	A1B3	A1B3	1	1
	**ARP59**		A1B2	0	1
	**ARP60**	A1B3	A1B3	1	1

DSM27421 (A2B3) × SSC120 (A1B2), basidium 2	**ARP61**	A2B3	A2B3	1	1
	**ARP62**		A2B2	1	1
	**ARP63**		A2B2	1	1
	**ARP64**	A2B3	A2B3	1	1
	**ARP65**	A2B3	A2B3	1	1
	**ARP66**		A2B2	1	1
	**ARP67**	A2B3	A2B3	1	1

DSM27421 (A2B3) × SSC129 (A1B1), basidium 1	**ARP68**	A1B1	A1B1	0	1
	**ARP70**	A1B1	A1B1	0	1
	**ARP71**	A1B1	A1B1	0	1

a Progeny selected for Illumina whole-genome sequencing are indicated in boldface.

b Strains without mating phenotype designations are sterile.

To further genotype and follow the mating type of each of the meiotic progeny, crosses of each were performed with the tester strains listed in Table 1 (results shown in Table 3). Sixteen of the progeny were sterile. All of the progeny from DSM27421 × SSC129 mated as mating type A1B1. The progeny from basidium 2 of DSM27421 × SSC120 either mated as A2B3 or were sterile, indicating that at least two genotypes were present in that progeny set. Similarly, the progeny from basidium 3 of DSM27421 × SSC120 either mated as A1B3 or were sterile, except one isolate (ARP56) that mated as A2B2. Thus, at least three genotypes (among four possible genotypes) were represented in this other progeny set. We identified the *MAT* alleles of the sterile progeny, using PCR-RFLP for the *STE3* and *HD* mating type-specific genes. The sterile progeny from basidium 3 of DSM27421 × SSC120 all had an A1B2 genotype, and the sterile progeny were all A2B2 in basidium 2 of the same cross.

The presence of multiple genotypes in the haploid progeny from two different basidia of a heterospecific cross is suggestive of random assortment during meiosis. However, the nature and frequency of meiotic recombination remained elusive. It was recently shown that the frequency of meiotic recombination in conspecific crosses of C. amylolentus is on average 1.59 crossovers/Mb and that all chromosomes have at least one crossover event (33). Because sequence divergence between recombining chromosomes of different species may decrease the rate of meiotic recombination due, for instance, to interference from the mismatch repair machinery that prevents recombination between diverged sequences (52–55), we sought to compare the findings in C. amylolentus with the meiotic recombination patterns in heterospecific crosses of C. amylolentus × DSM27421. To this end, paired-end Illumina sequencing was utilized to sequence the genomes of the C. amylolentus parents of the progeny collected, as well as those of a subset of the DSM27421 × C. amylolentus progeny. Specifically, we sequenced (i) two F1 progeny, SSA790 and SSB821, that were derived from the CBS6039 × CBS6273 conspecific cross; (ii) the F2 progeny recovered from cross SSA790 × SSB821 that could successfully mate with DSM27421 (i.e., SSC103, SSC120, SSC125, SSC129, and SSC130) (Fig. 5A) (Table 1); and (iii) 20 of the progeny derived from the heterospecific crosses of DSM27421 × SSC120 and DSM27421 × SSC129 (Tables 1 and 3).

To examine recombination at the genome-wide level and at high spatial resolution, we mapped the Illumina reads to either C. amylolentus CBS6039 or DSM27421 reference assemblies and generated plots for the distribution of single nucleotide polymorphisms (SNPs) along the length of each chromosome for each of the data sets. In this approach, crossovers along the chromosomes can be scored as transitions between haplotype segments of the two parental strains (Fig. 5B). As expected, at least 1 crossover was generally present on each chromosome of the C. amylolentus F1 progeny (SSA790 and SSB821), with a range of 1 to 6 crossovers. Additional crossovers were observed in the F2 progeny, and in six instances, no measurable recombination was detected (tracks enclosed by red lines in Fig. 5B). It should be noted, however, that crossovers between homozygous regions of the two interbred F1 strains (SSA790 and SSB821) are undetectable with this approach, resulting in lower estimates. Indeed, the crossover frequency estimated for the C. amylolentus F2 progeny was 1.32 crossovers/Mb on average, in comparison with 1.67 crossovers/Mb of the F1 progeny. Hence, considering both the C. amylolentus F1 and F2 progeny sets, the average of 1.42 crossovers/Mb across all chromosomes should be regarded as the very lowest estimate.

For the DSM27421 × SSC120 heterospecific cross, the SNP distribution revealed only five different genotypes (of 8 possible genotypes) among the 17 sequenced progeny derived from two different basidia (i.e., two independent occurrences of meiosis). Three genotypes were recovered from basidium 3 and only two from basidium 2 (Fig. 5C). The missing genotypes in each case most likely represented inviable meiotic products rather than biased sampling, as all spores that had germinated were analyzed (Tables 2 and 3). Furthermore, spores with similar genotypes in each basidium were mitotic clones that resulted from multiple rounds of mitosis to generate chains of spores following a single meiotic event (33, 56). Among the progeny representing unique genotypes, no recombination was detected in eight of the chromosomes (in a total of 14 cases; see the tracks enclosed by red lines in Fig. 5C). This was observed, for example, in 5 of 14 nuclear chromosomes of strain ARP56 (i.e., the 5 numbered 3, 8, 11, 12, and 13) and in three chromosomes of strain ARP61 (3, 10, and 14) (Fig. 5C). Nonetheless, at least one crossover per homeologous chromosome pair was detected in each of the two meiotic events analyzed. The range of the numbers of crossovers observed for the recombinant chromosomes was 1 to 5, and the crossover frequency along each chromosome ranged between 0.46 (chromosome 3) and 1.68 (chromosome 7), with an average of 0.97 crossovers/Mb, considering all of the chromosomes. Hence, the crossover frequency in the heterospecific crosses is on average lower than that estimated from C. amylolentus conspecific crosses (this study and reference 33).

Next, to test whether aneuploidy was present in the progeny set of both conspecific and heterospecific crosses, the relative chromosome copy numbers were determined from read counts. The nuclear chromosomes of all progeny had read depths that, relative to the average total read depth, were equal to about 1 (or equal to 0 after log2 transformation) (Fig. S8). However, a large segment of doubled read counts for chromosome 10 was observed for ARP45 and ARP60, indicating that the cell population of these strains included a significant number of *n* + 1 aneuploids. A third isolate (ARP44) appeared to be otherwise genotypically identical to ARP45 and ARP60 but was euploid. No other signs of aneuploidy were present in the sequenced progeny set (Fig. S1 and S8).

10.1128/mBio.00764-19.8 FIG S8 The progeny of C. amylolentus × DSM27421 are predominantly euploid. Read depth (binned in 5-kb nonoverlapping windows) was used to screen for chromosome aneuploidy. For each sequenced strain, read depth was normalized to the median read depth for that strain, log2-transformed, and plotted as a heat map with Circos. (A) All of the C. amylolentus parents used for crosses were euploid. (B) Two examples (ARP45 and ARP60) of *n* + 1 aneuploidy in the hybrid progeny are shown. Download FIG S8, PDF file, 1.2 MB. Copyright © 2019 Passer et al. 2019 Passer et al. This content is distributed under the terms of the Creative Commons Attribution 4.0 International license.

In addition to visualizing inheritance of nuclear DNA, SNP mapping was used to examine the inheritance of mitochondrial DNA (mtDNA). In crosses between individuals of different species or between extensively divergent populations of the same species, uniparental inheritance (UPI) of mitochondria may be altered due to a failure of the mechanisms governing UPI. This may lead to the generation of progeny with mtDNA from the parent that normally does not contribute (known as mtDNA leakage) and may facilitate recombination of mitochondrial genomes (57–59). Among the C. amylolentus F1 progeny, SSA790 (mating type A1B2) inherited its mtDNA from CBS6273 (A2B2), but SSB821 (A2B1) inherited its mtDNA from CBS6039 (A1B1) (Fig. 5B). On the other hand, all the C. amylolentus F2 progeny inherited their mtDNA from SSB821 (mating type A2B1), which is a pattern consistent with UPI and in line with previous studies in C. amylolentus (29). Likewise, the progeny derived from the DSM27421 × C. amylolentus heterospecific crosses all inherited mitochondrial DNA from DSM27421 (mating type A2B3) as assessed either by SNP mapping or by using a PCR-RFLP assay that was specific for the mitochondrial small-subunit (mitSSU) rRNA gene and that can distinguish between the two parental mitochondrial alleles (Table 3). The inheritance of CBS6039 mitochondria by SSB821 presumably represents an error in the execution of uniparental inheritance such as is occasionally observed among the members of a small population of the progeny in species with UPI of mitochondria (29, 60, 61).

Interestingly, the genome analysis of the three viable progeny from the DSM27421 × SSC129 cross (ARP68, ARP70, and ARP71) gave a different result (Fig. 5C). No crossovers were seen at any point in their genomes, and all nuclear DNA was inherited from SSC129. Conversely, all mitochondrial DNA was inherited from DSM27421. This finding indicates that karyogamy had not occurred for these three progeny but that the nuclear genome of one parent was combined with the mitochondrial genome of the other, a phenomena known as cytoduction (62).

Species that are products of recent divergence may still display signatures of continued exchange of genes (63–66). Given that DSM27421 can still produce viable progeny and undergo meiotic recombination with at least one strain of C. amylolentus, we searched for genomic signatures that could be suggestive of recent introgression between C. amylolentus and DSM27421 and CBS7118. Pairwise divergence was used as a proxy to search for evidence of the incorporation of DNA segments (regions of >5 kb) of a given strain into the genome of another strain. In this approach, putative introgressed regions would be detected as genomic tracts with nearly zero sequence divergence. Each genome was independently used as a reference to account for regions that could be missing in some of the strains. In all comparisons, the overall uniformity of sequence divergence across the genome (Fig. S9) suggested that there has been little, if any, recent introgression between the three lineages, which is consistent with the low spore viability of the tested heterospecific crosses.

10.1128/mBio.00764-19.9 FIG S9 Divergence plots showing no evidence of introgression between C. amylolentus, DSM27421, and CBS7118. Each plot represents the divergence *k* (with Jukes-Cantor correction) relative to (A) C. amylolentus CBS6039, (B) C. wingfieldii CBS7118, and (C) C. floricola DSM27421 (*y*-axis values represent percentages; *x*-axis values represent kilobases). Centromeres and *HD* and *P*/*R MAT* loci are highlighted as indicated in the key. Each data point represents the average divergence of itself plus two windows on each side. Download FIG S9, PDF file, 1.3 MB. Copyright © 2019 Passer et al. 2019 Passer et al. This content is distributed under the terms of the Creative Commons Attribution 4.0 International license.

**Taxonomic description of Cryptococcus wingfieldii and Cryptococcus floricola and phenotypic comparison with C. amylolentus.**

Description of *Cryptococcus wingfieldii* (van der Walt, Y. Yamada and N.P. Ferreira) Yurkov, A.R. Passer, M.A. Coelho, R.B. Billmyre, M. Nowrousian, M. Mittelbach, C.A. Cuomo, A.F. Averette, S. Sun, and J. Heitman, comb. nov. (MB 829726).

Basionym: *Sterigmatomyces wingfieldii* Van der Walt, Y. Yamada and N.P. Ferreira, Antonie van Leeuwenhoek **53**:138, 1987 (MB 133483).

Holotype: PREM 48490 in the Herbarium for Fungi of the Research Institute for Plant Protection, Pretoria, South Africa.

Ex-type cultures: CBS 7118, PYCC 5373, JCM 7368, NRRL Y-17143, DSM 107903. (Physiological characteristics of the species are provided in reference 67).

The species is known from a single culture isolated from insect frass in South Africa. This yeast was originally described in the genus *Sterigmatomyces* and later transferred to the monotypic genus *Tsuchiyaea* as Tsuchiyaea wingfieldii (discussed in references 68 and 38). Phylogenetic analyses suggested its close relationship with Cryptococcus amylolentus, so Tsuchiyaea wingfieldii was considered to be a synonym of C. amylolentus (38, 68). Results obtained in the present study suggest that Cryptococcus amylolentus, Tsuchiyaea wingfieldii, and the newly described Cryptococcus floricola (see below) represent genetically isolated species.

Description of Cryptococcus floricola Yurkov, A.R. Passer, M.A. Coelho, R.B. Billmyre, M. Nowrousian, M. Mittelbach, C.A. Cuomo, A.F. Averette, S. Sun and J. Heitman, sp. nov. (MB 829727).

Etymology: The species epithet “floricola” refers to its origin of isolation, flower nectar.

**FIG 6:**
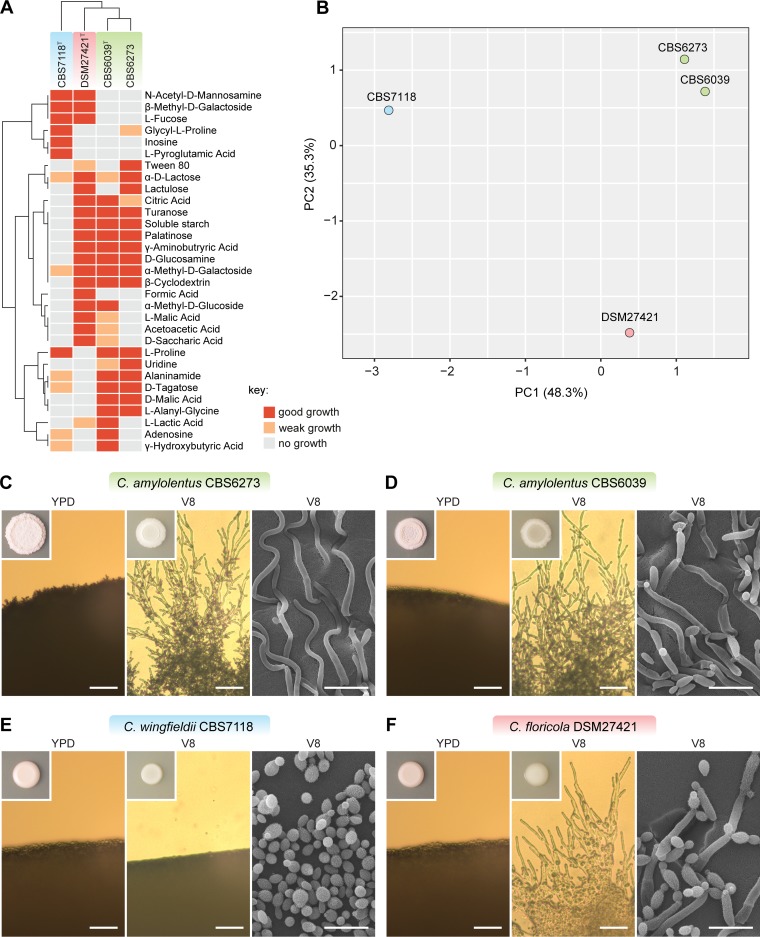
Phenotypic and morphological characteristics distinguishing C. amylolentus, C. floricola, and C. wingfieldii. (A) The ability of strains of C. amylolentus, C. floricola, and C. wingfieldii to oxidize and assimilate different carbon sources, assayed using Biolog YT, FF, and GEN III MicroPlates, is displayed as a heat map. No scaling is applied to rows. Both rows and columns are clustered using correlation distance and average linkage. (B) Principal-component analysis showing the similarity of growth responses of strains of C. amylolentus, C. floricola, and C. wingfieldii,assayed using Biolog YT, FF, and GEN III MicroPlates. Percentages of the total variances described by the first two extracted factors are given on the axes. (C to F) Macroscopic and microscopic morphology is shown for (C) C. amylolentus CBS6273, (D) C. amylolentus CBS6039, (E) C. wingfieldii CBS7118, and (F) C. floricola DSM27421. Colonies were grown for 1 week on YPD or V8 media (inset images) and imaged by light microscopy (left two segments of each panel; scale bars = 50 μm) and by SEM (right segment of each panel; scale bars = 10 μm). V8 medium promoted hyphal growth, except for strain CBS7118, which grew only as a yeast.

After growth on 5% malt agar (MA) plates for 2 weeks at 25°C, the streak culture was mucoid, smooth, and cream in color and was partially transparent with a glistening surface. Upon aging, the colony turned dull and tan and appeared wrinkled. After growth on yeast malt (YM) agar plates for 7 days at 25°C, cells were ellipsoidal, fusoidal, and elongate to cylindrical (4 to 10 × 3 to 5 μm); occurred singly; and proliferated by polar budding (Fig. 6). Pseudohyphae and true hyphae were not observed after 1 month on Dalmau plate culture on potato-dextrose agar (PDA) and corn meal agar (CMA) at 16 to 22°C. Chlamydospore-like cells were observed in older cultures (3 to 4 weeks of age) on YM agar and PDA under conditions of incubation at 16°C. Ballistospores were not observed.

Sexual reproduction with compatible strains of Cryptococcus amylolentus was observed on V8 agar (pH 5) after growth for 1 week in darkness at room temperature (22°C to 24°C). Fused clamp cells were present. Aerial hyphae were produced. The tips of the aerial hyphae formed basidia (3 to 6 × 4 to 6 μm in size) with four parallel spore chains of budding globose basidiospores (1.6 to 2 × 2 to 2.5 μm in size) arising from the apical surface of basidia.

Assimilation of carbon compounds: growth on d-glucose, d-galactose, l-sorbose, d-glucosamine, d-ribose, d-xylose, l-arabinose, d-arabinose, l-rhamnose, sucrose, maltose, trehalose, α-methyl-d-glucoside, cellobiose, salicin, melibiose, lactose, raffinose, melezitose, soluble starch, glycerol, erythritol, ribitol, xylitol, l-arabinitol, d-glucitol, d-mannitol, myo-inositol, 2-keto-d-gluconate, d-gluconate, d-glucoronate, d-galacturonate, succinate, citrate, ethanol, palatinose, l-malic acid, and gentiobiose. No growth occurred on inulin, galactitol, dl-lactate, methanol, quinic acid, d-glucarate, galactaric acid, Tween 40 and Tween 80, and nitrate and nitrite.

Urea hydrolysis and diazonium blue B (DBB) reaction results were positive. Growth results in 50% and 60% d-glucose were positive. Growth results in the presence of 1%, 4%, 5%, 8% and 10% NaCl were positive. The maximum growth temperature was 30°C.

Molecular characteristics (type strain): nucleotide sequences of ITS-LSU (D1/D2 domains) rRNA have been deposited in NCBI/EMBL (GenBank).

Deposits: holotype MoM 837 isolated from nectar of Echium leucophaeum in Tenerife, Canary Islands, Spain (28°33.80'N, 16°17.41'W), preserved in a metabolically inactive state at the German Collection of Microorganisms and Cell Cultures, Braunschweig, Germany, as DSM 27421^T^. Ex-type cultures are deposited in the CBS yeast collection of the Westerdijk Fungal Biodiversity Institute, Utrecht, The Netherlands (CBS 15421) and the Portuguese Yeast Culture Collection (PYCC), Caparica, Portugal (PYCC 8315).

Notes: the species differs from closely related species Cryptococcus amylolentus and Cryptococcus wingfieldii in the ability to grow on d-glucosamine, α-methyl-d-glucoside, β-methyl-d-glucoside, citric acid, and some aldaric acids such as d-malic, d-saccharic, d-tartaric, l-malic, l-tartaric, and mucic acids (Fig. 6). Additional isolates of the three species can be identified using partial nucleotide sequences of *TEF1* and *RPB1* genes, which showed 97% to 98% similarity in pairwise comparisons.

## DISCUSSION

Speciation requires the establishment of reproductive isolation and thus the cessation of gene flow between genetically diverged lineages. With the advancement of genome sequencing techniques, it has become less challenging to obtain data on whole-genome sequences and to compare levels of genetic and genomic divergence between closely related species. This is particularly true for eukaryotic microbes, such as fungi, with relatively small and simple genomes. However, it remains less straightforward to obtain the complete view of the reproductive compatibility between diverging lineages that could represent cryptic or nascent species. This could be due to several factors, including the following: (i) environmental cues for mating can differ among closely related fungal species, and the conditions that induce sexual reproduction between different species can thus be difficult to reconstitute in the laboratory; and (ii) even in cases where mating structures are observed between potential different species, the resulting progeny may have reduced viability. It is thus important to assess the viability of the mating progeny, which are sometimes difficult to recover but nevertheless are the true indicators of whether reproductive barriers are present or absent.

In our study, we observed 93.5% to 94.4% pairwise genome-level sequence similarity among the genomes of C. amylolentus CBS6039, C. wingfieldii CBS7118, and C. floricola DSM27421. These values are slightly higher than but generally comparable to those observed among the species in the C. neoformans/C. gattii species complex (69, 70). This finding is also consistent with the observed chromosomal rearrangements among these species, where, although inversions have been identified, they are generally small and simple and concentrated in the subtelomeric regions, and we identified only one balanced translocation that is shared by isolates CBS7118 and DSM27421. In contrast, multiple chromosomal translocations, as well as large and complex inversion/transposition regions, have been identified among species in the C. neoformans/C. gattii complex (10, 41). Interestingly, the breakpoint of the translocation identified in CBS7118 and DSM27421 is located within the centromeric region, suggesting that it might represent the result of intercentromeric ectopic recombination mediated by shared transposable element present in the centromeres, which have been shown in previous studies to play important roles in the genome evolution of the members of the C. neoformans/C. gattii complex as well as that of their most closely related species (33).

Comparisons of the *MAT* loci showed that the *P*/*R* locus region in each of C. wingfieldii CBS7118 and C. floricola DSM27421 has undergone further rearrangements compared to C. amylolentus CBS6039 and CBS6273, with respect to regions both within and flanking the *MAT* regions. However, the *P*/*R* alleles of the same mating type in different species are still more similar to each other than to those of the opposite mating types in the same species, which is consistent with observations in other basidiomycetes species (48–51, 71).

Our analyses also suggested that while the same transposable elements are enriched in the centromeric regions of all four isolates analyzed, C. wingfieldii CBS7118 and C. floricola DSM27421 have additional copies of these transposable elements, leading to the presence of predicted centromeres that are larger than those of C. amylolentus isolates (Fig. S4). The primary function of the centromere is to generate a functional kinetochore for faithful chromosome segregation, and, as a few studies have suggested (72, 73), differences in centromere structure may mediate or reinforce hybrid incompatibility, thus facilitating speciation. In C. neoformans, centromeres can undergo expansion/contraction, likely through recombination mechanisms involving transposable elements (42). Thus, it is possible that C. wingfieldii and C. floricola have evolved to sustain larger centromeres. Whether or not the relatively large centromeres of C. floricola have expanded centromeric heterochromatin relative to the smaller C. amylolentus centromeres that could possibly lead to unequal tension on centromeres remains unknown but will be an interesting topic to explore in future studies.

Taken together, our genomic comparison analyses collectively suggest that isolates CBS7118 and DSM27421 represent distinct species (C. wingfieldii and C. floricola, respectively) that, along with C. amylolentus, form the sister clade to the pathogenic C. neoformans/C. gattii species complex.

In our study, C. wingfieldii (CBS7118) did not mate with C. floricola or with any of the C. amylolentus strains. As mentioned earlier, this could be due to our inability to recapitulate the ideal conditions for C. wingfieldii to undergo sexual reproduction. Alternatively, it could be that C. wingfieldii has already undergone a level of divergence sufficient to ensure that prezygotic reproductive barriers have already been established between C. wingfieldii and the other two species, C. amylolentus and C. floricola. If that is the case, this is not yet reflected at the level of the pheromone genes, which are identical between C. wingfieldii and C. amylolentus CBS6039 (Fig. S6). Another possibility could be that cell-cell fusion still occurs between C. wingfieldii and C. amylolentus but that the downstream sexual development governed by the *HD* genes is compromised; thus, no clear signs of mating (e.g., filamentation) could be detected. On the other hand, we observed successful sexual reproduction between C. floricola DSM27421 and the C. amylolentus tester strains with various genetic backgrounds, with mating structures highly similar to those observed in crosses between C. amylolentus isolates (29). Importantly, we were able to successfully recover mating progeny from these crosses. However, when the spores were dissected and analyzed, the progeny from crosses between C. floricola and C. amylolentus showed a highly reduced germination rate, indicating that most of the sexual progeny were inviable. Similarly low levels of spore viability in crosses between sister species of the C. neoformans/C. gattii complex were reported previously (10, 11). This suggests that postzygotic instead of prezygotic reproductive isolation has been established between C. floricola and C. amylolentus.

Postzygotic isolation in this case could have resulted from compromised meiosis due to the elevated levels of genetic divergence, as well as due to the presence of chromosomal rearrangements such as translocations and inversions. Together, these differences could have reduced the frequency of crossing-over and resulted in chromosome missegregation, thus leading to the presence of unbalanced meiotic products. Consistent with this hypothesis, in analyses of progeny from crosses between C. amylolentus and C. floricola, we found that crossovers occurred at a lower frequency than in crosses between C. amylolentus isolates (33). Specifically, we observed no signs of crossover in several chromosomes, which likely resulted from the presence of chromatids that did not participate in crossover during meiosis I, in similarity to observations reported for crosses within the C. neoformans/C. gattii species complex (74, 75). Alternatively, the reduced spore viability could have been due to the presence of mechanisms such as Bateson-Dobzhansky-Muller incompatibility that have evolved between the diverging lineages. It should be noted that the reduction of spore viability resulting from crosses between C. amylolentus and C. floricola appears to have been less severe than that seen in the case of interspecific crosses in the C. neoformans/C. gattii complex, suggesting that speciation events among C. amylolentus and C. floricola likely occurred more recently.

The isolates representing C. amylolentus, C. floricola, and C. wingfieldii have been isolated from different geographic areas. It is possible that geographic isolation further enhances genetic isolation among these species (19). A combination of genetic incompatibility and differences in distribution range and dispersal vectors could explain the lack of mating between these species. Note that all isolates of the C. amylolentus species complex were found in habitats associated with insects, including insect frass and floral nectar. Although C. amylolentus and C. wingfieldii have so far been found only in South Africa, their dispersal may depend on different vectors. In contrast to the other two species, C. floricola was isolated much farther away, on the Canary Islands (Macaronesia), and may thus have a different range. The three species show distinctive phenotypic characteristics, and principal-component analyses (PCA) of the physiological profiles of the three species classified them into three distinct groups (Fig. 6A). For example, it appears that C. floricola may have a unique ability to grow under high-glucose conditions, which could have been selected for, as C. floricola was originally found to be associated with flower nectar. It would be difficult to dissect whether the observed divergent physiological features in these species are the causes or the consequences of their ecological separation leading to or facilitating genetic isolation and, eventually, the establishment of reproductive isolation between closely related nascent species.

Currently, each of the three species described in our study is represented by a limited number of isolates: two are known for C. amylolentus and one each for C. wingfieldii and C. floricola. Our studies revealed each represents a distinct, well-defined species, with no detectable introgression or hybridization between genomes. Our analyses also indicate that these are rare species and may have more restricted geographic niches and nutritional requirements, which could have hindered their successful isolation from environmental samples. For instance, the C. floricola isolate was found among another 220 strains isolated from 480 sampled flowers (37). With continuing advances in sampling and identification techniques, it is possible that additional isolates of these species will become available in the future, thereby enabling population-based studies and further analysis of modes of sexual reproduction. For example, we have shown that the protein-coding genes *TEF1* and *RPB1* represent promising DNA markers that can be used to differentiate species closely related to the C. amylolentus species complex. Our definition of these isolates as species is in accord with previous studies of these and other species in which a limited number of isolates, or a single isolate, was sufficient for defining and naming novel taxa (67, 76–78).

The species C. amylolentus, C. floricola, and C. wingfieldii form a tight clade that is closely related to the major human-pathogenic fungal C. neoformans/C. gattii complex, the members of which collectively cause over 200,000 cases annually (27). Understanding and dissecting the genetic basis and evolutionary events that resulted in the species in this complex becoming successful human-pathogen species require an analysis and comparison of the genomes of not only these pathogens but also those of their nonpathogenic close relatives. It was shown that C. amylolentus was not virulent in a murine model (28), although it might be virulent in insect models (79). Both C. floricola and C. wingfieldii show no growth at elevated temperatures mimicking the body temperatures of mammals, suggesting that they are also likely avirulent in humans. Thus, these three species provide a unique opportunity to gain further insights into the evolution of the species in the pathogenic C. neoformans/C. gattii species complex.

## MATERIALS AND METHODS

### Mating crosses, spore dissection, and genotyping.

Plates of V8 (pH = 5) mating medium (80) were inoculated with suspensions of yeast cells in water. For each pairwise cross, 10 μl of each strain was pipetted onto the same spot of medium. The plates were then incubated in the dark at 24°C for at least 1 week and checked periodically thereafter. Mating responses were observed with a compound light microscope, and results were scored as positive if aerial hyphae, basidia, and spores were observed. Spore germination was quantified by using a micromanipulator to transfer individual basidia from mating plates to yeast extract-peptone-dextrose (YPD) plates and then to separate individual spores into a defined grid. The plates were incubated at 24°C to allow colonies to form. The proportion of spores that germinated was calculated for each basidium by dividing the number of spores that formed a colony by the number of spores that were plated. The mating type of the viable progeny was determined by crossing them with tester strains (indicated in Table 1). For the sterile progeny, the mating genotype was determined via a PCR-RFLP assay using the primers listed in Table S4 at https://figshare.com/s/80a83fe2c088854e7dee targeting a small region of the *P*/*R* or *HD* locus, and then digestion was performed using restriction enzymes (BsaHI and Tsp45I for *P*/*R* and *HD* loci, respectively) that yielded a restriction pattern unique to each *MAT* alleles. A similar approach was used to identify different alleles of the mitochondrial small-subunit rRNA using MseI.

### Microscopy.

Inoculation and incubation conditions for microscopy specimens were the same as for the other mating crosses. For the light micrographs, a cross of DSM27421 × CBS6039 on V8 (pH = 5) mating medium was incubated for 39 days. The crosses were viewed with a Zeiss Scope.A1 microscope and photographed with a Zeiss Axiocam 105 color camera. For the scanning electron micrographs (SEM), a cross of DSM27421 × CBS6039 on V8 (pH = 5) mating medium was incubated for 11 days. SEM was performed at the North Carolina State University Center for Electron Microscopy, Raleigh, NC, USA. Agar blocks (approximately 0.5 cm^3^) containing hyphae on the edges of mating patches were excised and fixed in 0.1 M sodium cacodylate buffer (pH = 6.8) containing 3% glutaraldehyde at 4°C for several weeks. Before viewing, the agar blocks were rinsed with cold 0.1 M sodium cacodylate buffer (pH = 6.8) three times and then dehydrated in a graded series of ethanol to reach 100% ethanol. The blocks were subjected to critical-point drying with liquid CO_2_ (Tousimis Research Corp.) and sputter coated with 50 Å of gold/palladium using a Hummer 6.2 sputter coater (Anatech USA). The samples were viewed at 15 kV with a JSM 5900LV scanning electron microscope (JEOL) and captured with a Digital Scan Generator (JEOL) image acquisition system.

### DNA extraction and genome sequencing of DSM27421 and CBS7118 strains.

Genomic DNA was extracted using a modified cetyltrimethylammonium bromide (CTAB) protocol as previously reported (42). High-molecular-weight DNA samples were obtained by spooling out the precipitated DNA using a glass rod instead of centrifugation. Genomic DNA size and integrity were confirmed by CHEF electrophoresis as previously described (33, 42). Sequencing of the DSM27421 and CBS7118 genomes was carried out using Illumina, Pacific Biosciences (PacBio), and Oxford Nanopore (ONT) technologies. For Illumina sequencing of the CBS7118 genome, two libraries were constructed with average insertion sizes of 187 bases and 1.9 kb (jumping library). For the fragment library, 100 ng of genomic DNA was sheared to ∼250 bp using a Covaris LE instrument and was prepared for sequencing as previously described (81). The ∼2-kb jumping library was prepared using a 2-to-5-kb-insertion Illumina mate-pair library prep kit (V2; Illumina) as previously described (82). These libraries were sequenced by the use of the Broad Institute Genomics Platform on an Illumina HiSeq 2000 system to generate paired 101-base reads. The genome of DSM27421 was sequenced from a small (∼350-bp)-insertion-size library on a HiSeq 2500 system to generate 151 base reads. For PacBio sequencing, large (15-to-20-kb)-insertion-size libraries were generated and run on a PacBio RS II or Sequel (2.0 chemistry) system(s) (see Table S1 at https://figshare.com/s/80a83fe2c088854e7dee). PacBio sequencing and Illumina sequencing of DSM27421 were performed at the Sequencing and Genomic Technologies Core Facility of the Duke Center for Genomic and Computational Biology. Nanopore sequencing was performed per the manufacturer's guidelines. Libraries were prepared by the use of a SQK-LSK108 one-dimensional (1D) ligation sequencing kit and were run for 48 h in R9 flow cells (FLO-MN106) using a MinION system. MinION sequencing and live base-calling were controlled using Oxford Nanopore Technologies MinKNOW v.1.10.16 software.

### Genome assembly and gene prediction of strains DSM27421 and CBS7118.

The initial assembly of CBS7118 was generated from approximately 100× reads from the fragment library and 50× reads from the jumping library using ALLPATHS-LG (83) version R47093. The resulting assembly consisted of 83 scaffolds and 196 contigs. For DSM27421, the initial assembly was generated using SPAdes v3.10 (84), resulting in 573 scaffolds and 636 contigs. Improved assemblies were generated using both PacBio and Nanopore long-read read data with Canu v1.7 (85) and the default parameters and an estimated genome size of 20 Mb. Because the read length profiles of the sequencing runs differed considerably (see Table S1 at https://figshare.com/s/80a83fe2c088854e7dee), different read length combinations were tested as input for Canu. For DSM27421, the final draft assembly was generated by combining the read data from two PacBio runs (reads above 10 kb from run 1 and all reads from run 2) and the read data from the ONT reads above 10 kb (run 1) (see Table S1 at https://figshare.com/s/80a83fe2c088854e7dee). For the CBS7118 strain, combining the ONT and PacBio reads resulted in more-fragmented assemblies than using the PacBio data alone, most likely due to a shorter read length and higher noise level of the ONT reads; thus, only PacBio reads were used (see Table S1 at https://figshare.com/s/80a83fe2c088854e7dee). Genome assembly statistics and additional genomic features of each strain are reported in Table S1 at https://figshare.com/s/80a83fe2c088854e7dee. The accuracy of the resulting assemblies was improved by correcting errors using five rounds of Pilon (v1.22) polishing (‘–fix all’ setting) (86) and the Illumina reads mapped to the respective assemblies by the use of BWA-MEM (v0.7.17-r1188) (87). Gene models were predicted *ab initio* using MAKER v2.31.18 (88) with predicted proteins from Cryptococcus neoformans H99 (41) and Cryptococcus amylolentus CBS6039 (33) as inputs.

### Whole-genome pairwise identity and phylogenetic analyses.

To calculate average pairwise identities (Fig. 1C), genomes were aligned using NUCmer (v3.22) from the MUMmer package (89) with parameter ‘-mum.’ Alignments were filtered with a delta filter using parameters ‘-1’ to select 1-to-1 alignments allowing for rearrangements and ‘-l 100’ to select a minimal alignment length of 100 bases. To confirm the phylogenetic placement of C. amylolentus species complex within the *Cryptococcus* lineage, a maximum likelihood (ML) phylogram was inferred from a concatenated alignment of the internal transcribed spacer region (ITS1, 5.8S and ITS2), *RPB1* and *TEF1* genes. Available sequences were obtained from GenBank, and additional sequences were determined by Sanger sequencing and deposited in GenBank (see Table S5 at https://figshare.com/s/80a83fe2c088854e7dee). The individual gene sequences were aligned by MAFFT (v7.245) (90) using the E-INS-i algorithm and were subsequently concatenated. A maximum likelihood phylogram was generated for this data set using MEGA v6.06 (91) (with the parameters uniform rates, complete gap deletion, subtree pruning and regrafting level 5, very weak branch swap filter, and BioNJ initial tree) and the Kimura 2-parameter substitution model (92). Phylogram stability was measured with 1,000 bootstrap replicates.

For finer resolution of the C. amylolentus species complex, orthologs were identified among C. wingfieldii (CBS7118), C. floricola (DSM27421), and C. amylolentus (CBS6039 and CBS6273) and an outgroup (C. depauperatus CBS7855) based on BLASTP pairwise matches with expected values of <1e−5 using ORTHOMCL (v1.4). A phylogeny was inferred from 4,896 single-copy genes as follows. Individual proteins were aligned using MUSCLE (93), the individual alignments were concatenated, and poorly aligning regions were removed with trimAl (94). This sequence was input to RAxML (v8.2.4; raxmlHPC-PTHREADS-SSE3) (95) and a phylogeny estimated in rapid bootstrapping mode with model PROTCATWAG and 1,000 bootstrap replicates. To estimate the level of gene support for the best tree obtained from this analysis, the individual gene trees were inferred from protein alignments using RAxML with the same settings. The subsets of gene trees with at least 50% bootstrap support at all nodes were input to RAxML with the best tree to estimate gene support frequency (GSF) and internode certainty (IC) at each node (39, 40) using settings -f b and -f I, respectively.

### Analysis of genomic features and synteny comparison.

Repetitive DNA content, including transposable elements, was analyzed with RepeatMasker (RepeatMasker Open-4.0. 2013–2015; http://www.repeatmasker.org), using REPBASE v23.09 (96), and with TransposonPSI (http://transposonpsi.sourceforge.net/). Centromeres were predicted upon detection of centromere-associated LTR elements previously reported in C. amylolentus (Tcen1 to Tcen6) (33) and C. neoformans (Tcn1 to Tcn6) (41, 97). These elements were identified from the RepeatMasker output and confirmed by BLASTN using the C. neoformans sequences as query. Most of these elements mapped to the largest open reading frame (ORF)-free region in each contig, including one end of contigs 7a and 7b, which represent arms of the same chromosome, in the DSM27421 assembly. Centromere locations were further refined by mapping onto the DSM27421 and CBS7118 assemblies the position of each of the centromere flanking genes previously identified in C. amylolentus (33), using BLAST analyses. The final centromere length was measured as the intergenic region between the centromere flanking genes (see Table S3 at https://figshare.com/s/80a83fe2c088854e7dee) and subsequently compared to the previously reported centromere lengths of C. neoformans H99, C. deuterogattii R265, and C. deneoformans JEC21 (42) (Fig. S5). Statistical tests (Tukey-Kramer honestly significant difference [HSD] tests) were performed using JMP Pro 13 (SAS Institute). The GC content was calculated in nonoverlapping 5-kb windows using a modified perl script (gcSkew.pl; https://github.com/Geo-omics/scripts/blob/master/scripts/gcSkew) and plotted as the deviation from the genome average for each contig. rRNA genes (18S, 5.8S, 25S, and 5S) and tRNA genes were inferred and annotated using RNAmmer (v1.2) (98) and tRNAscan-SE (v2.0) (99), respectively. Telomeric repeats were identified using the EMBOSS fuzznuc function (100) based on known telomere repeat sequences of C. amylolentus. A search pattern of 2 × C(3,4)GCTAAC was used, allowing for minor variation between the repeats.

Synteny comparisons across the genomes of all strains were conducted using megablast (word size: 28), and the results were plotted, together with the other genomic features, using Circos (v0.69-6) (101) (as shown in Fig. 3; see also Fig. S3A and S3B). Additional whole-genome alignments were conducted with Satsuma (https://github.com/bioinfologics/satsuma2) (102) with the default parameters, and the output was sequentially passed to the visualization tools “BlockDisplaySatsuma” and “ChromosomePaint” included in the same package to generate a postscript file. Centromere and other genomic features were superimposed at scale in the final figure (shown in Fig. S3C to E) based on their respective genome coordinates. Linear synteny comparisons (Fig. S4; see also S5A) were generated with the Python application Easyfig (103) and the following settings: -svg -f gene frame -legend both -leg_name locus_tag -blast_col 230 230 230 230 230 230 -blast_col_inv 241 209 212 241 209 212 -bo F -width 5000 -ann_height 450 -blast_height 300 -f1 T -f2 10000 -min_length 1000.

### CHEF analysis and chromoblots.

CHEF gel electrophoresis and chromoblot analyses were carried out as described in a previous publication (28). Probes that hybridized to each chromosome arm were generated by PCR using the primers listed in Table S4 (posted at https://figshare.com/s/80a83fe2c088854e7dee).

### Analysis of mating type regions.

*MAT* regions were identified by BLAST searches against the well-annotated *MAT*-derived proteins from C. neoformans and were manually reannotated if necessary. Synteny between *MAT* regions of different strains was based on bidirectional BLAST analyses of the corresponding predicted proteins. The short pheromone precursor genes (or their remnants) were not always found among the predicted genes and were thus identified manually.

### Read mapping, variant calling and filtering, aneuploidy, and genome-wide recombination.

The genomes of C. amylolentus F1 and F2 progeny, and the genomes of the progeny derived from DSM27421 × C. amylolentus heterospecific crosses, were subjected to Illumina paired-end sequencing on a HiSeq 4000 system. Read lengths were 100 or 150 bases, depending on the run. Genomic signatures consistent with meiotic recombination and aneuploidy were inferred, respectively, from the SNP distribution and the read depth obtained for each chromosome. Paired-end reads of C. amylolentus F1 and F2 progeny were mapped to the C. amylolentus CBS6039 reference genome, whereas those resulting from DSM27421 × C. amylolentus crosses were mapped to the newly generated DSM27421 assembly. In both cases, reads were mapped using the BWA-MEM short-read aligner (v0.7.17-r1188) with default settings. SNP discovery, variant evaluation, and further refinements were carried out with the Genome Analysis Toolkit (GATK) best-practices pipeline (104) (v4.0.1.2), including the use of Picard tools to convert SAM to sorted BAM files, fix read groups (module: ‘AddOrReplaceReadGroups’; SORT_ORDER=coordinate), and mark duplicates. Variant sites were identified with HaplotypeCaller from GATK using the haploid mode setting, and only high-confidence variants that passed a filtration step were retained (the “VariantFiltration” module used the following criteria: DP < 20 ǁ QD < 15.0 ǁ FS > 60.0 ǁ MQ < 55.0 ǁ SOR > 4.0). Finally, filtered variants found in each contig/chromosome were binned into 5-kb windows and parsed into a tab-delimited format to allow visualization in Circos. Recombination tracts are observed as transitions between haplotype segments from the two parental strains along the chromosomes. Read count data were used to screen for gross aneuploidy of chromosomes. First, reads were counted in 5-kb nonoverlapping windows using the module “count_dna” from the Alfred package (v0.1.7) (https://github.com/tobiasrausch/alfred) and the BAM file obtained after read mapping as the input. Then, the resulting read counts were subjected to median normalization and log2 transformation and were finally parsed into a tab-delimited format and plotted as a heat map in Circos.

### Divergence plots.

To detect regions of introgression (>5 kb), Illumina reads generated for each of the strains were processed with the methods described above for alignment to each of the reference genome assemblies (C. amylolentus CBS6039, DSM27421, or CBS7118). The resulting consensus genotype in the variant call format was converted to the FASTQ format by limiting the maximum depth value to 200 to avoid overrepresented regions. A FASTA file was then generated in which bases with quality values lower than 20 (equivalent to 99% accuracy) were soft-masked to lowercase and ambiguous bases were subsequently converted to an “N.” Levels of divergence per site (*k*, with Jukes-Cantor correction) between pairs of strains were estimated in VariScan v.2.0.3 (105) using a nonoverlapping sliding-window of 5,000 sites. For easier visualization and interpretation, each data point presented in Fig. S9 represents an average of values corresponding to itself and two filtered windows on either side. Because highly divergent regions are challenging to align to a reference genome, all divergence estimates should be regarded as minimum estimates.

### FACS analysis.

To determine the ploidy of the full set of strains used in this study (Fig. S1), isolates were cultured on YPD medium for 2 to 4 days at 25°C and processed for flow cytometry as previously described (106) but without sonication. For each sample, approximately 10,000 cells were analyzed on the FL1 channel on a Becton, Dickinson FACScan apparatus at the Duke Cancer Institute Flow Cytometry Shared Resource. C. deneoformans JEC21 (Dα) and C. deneoformans XL143 (αDDα) were used as haploid and diploid controls, respectively.

### Physiological tests.

Phenotype microarray testing of carbon sources was examined using the Biolog MicroStation and YT, FF, and GEN III MicroPlates following the manufacturer's instructions (Biolog Inc., Hayward, CA, USA). Yeasts were incubated on potato dextrose agar (PDA; Difco) at room temperature. Yeast biomass was harvested from PDA and suspended in IF-B inoculation solution (Biolog Inc., Hayward, CA, USA), and the turbidity was adjusted to the transmittance value provided by the manufacturer. MicroPlates were sealed to prevent desiccation and were incubated at room temperature, and levels of growth were measured after 1, 2, 3, and 4 weeks, with the optical density recorded at 590 and 750 nm. The ability to utilize particular substrates by individual strains was recorded as positive, weak, or negative.

Analyses of utilization of d-glucosamine, maltose, methyl-alpha-d-glucoside, melezitose, soluble starch, d-glucitol, and galactitol and of dl-lactic, succinic, citric, and aldaric acids were performed in 3.5 ml of liquid media according to commonly used protocols (107, 108). Growth tests in the presence of 50% glucose, 60% glucose, 5% NaCl, 8% NaCl, and 10% NaCl was were performed in 3.5 ml liquid media according to commonly used protocols (108).

A total of 156 growth responses were recorded with YT, FF, and GEN III MicroPlates. Invariable results were discarded, and results from 31 variable tests were visualized with a heat map and subjected to principal-component analysis (PCA) using the ClustVis Web tool (109). Growth response results were assigned values of 1 for positive growth, 0.5 for weak growth, and 0 for negative growth. The following settings were used for the analysis: singular-value decomposition (SVD) method with imputation; no transformation; no row scaling; no row centering. Also, constant columns were removed. A heat map was produced using the following settings: no scaling was applied to rows, and both rows and columns were clustered using correlation distance and average linkage. PCA was performed with the following settings: no scaling was applied to rows, and SVD with imputation was used to calculate the principal components.

### Data availability.

Nucleotide sequences of ITS-LSU (D1/D2 domains) rRNA have been deposited in NCBI/EMBL (GenBank) under accession number HG421442. Sequencing reads for C. floricola DSM27421 (BioProject PRJNA496466) and C. wingfieldii CBS7118 (BioProject PRJNA496468) and for the progeny of crosses (BioProject PRJNA496469) are available in the NCBI SRA database. The DSM27421 and CBS7118 genome assemblies have been deposited at DDBJ/ENA/GenBank under accession numbers RRZH00000000 and CP034261 to CP034275, respectively. Illumina assemblies of CBS7118 are available under BioProject PRJNA200567. Other sequence accession numbers are listed in Table S5 (posted at https://figshare.com/s/80a83fe2c088854e7dee).
