# Supplementary material for: Genetic and Genomic Analyses Reveal Boundaries between Species Closely Related to Cryptococcus Pathogens
Source: mBio. 2019 Jun 11;10(3):e00764-19. doi: 10.1128/mBio.00764-19 (PMC6561019; doi:10.1128/mBio.00764-19)
Supplement: FIG S1 [file mBio.00764-19-sf001.pdf]

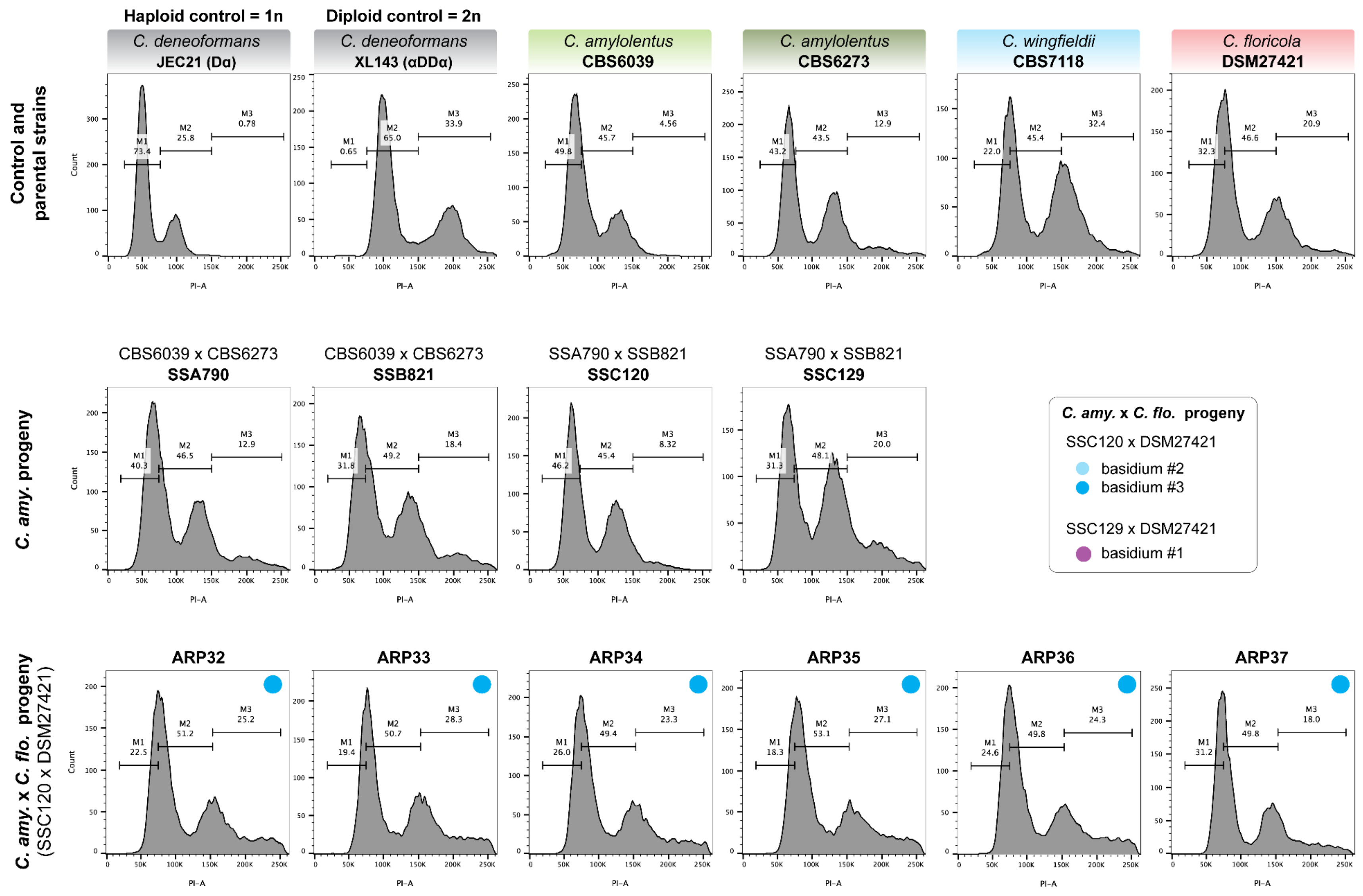

Fig. S1. Ploidy determination by FACS for strains used in this study. *C. deneoformans* JEC21 (Dα) and *C. deneoformans* XL143 (αDDα) were used as haploid and diploid controls, respectively. Approximately 10,000 cells were analyzed. Propidium iodide area (PI-A) is shown in the x-axis.

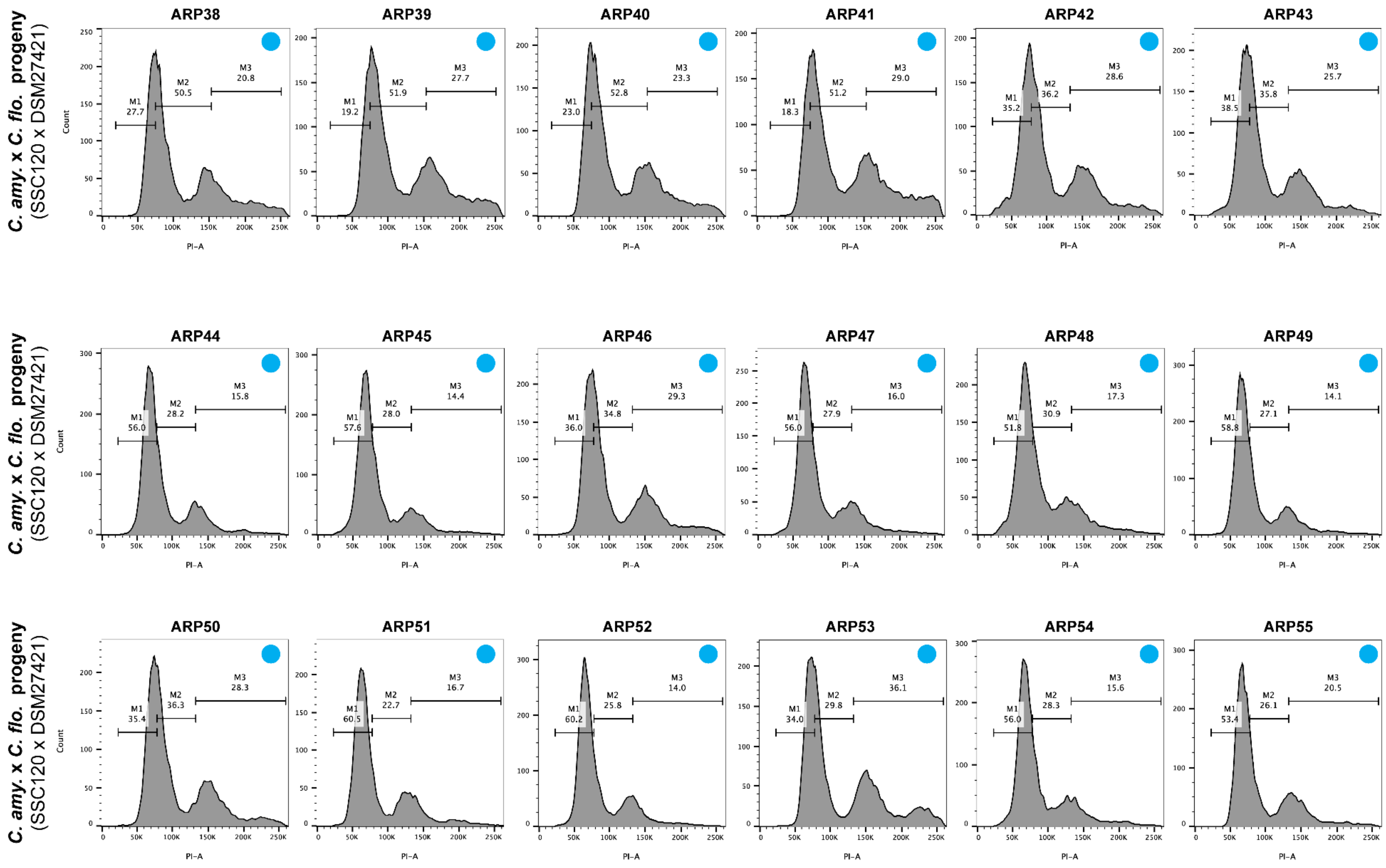

Fig. S1. Continued.

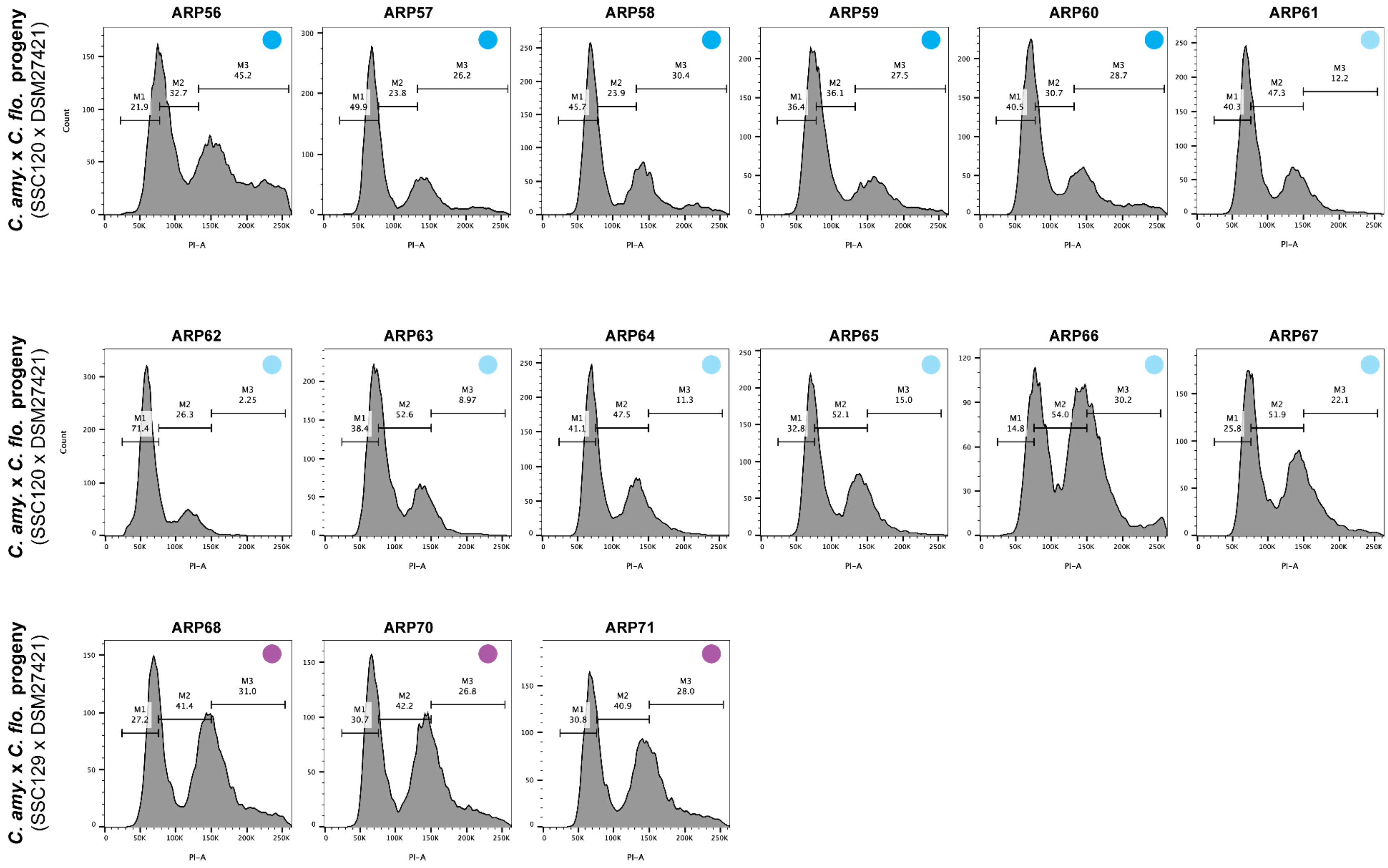

Fig. S1. Continued.
