# Supplementary figures and images for: Genetic and Genomic Analyses Reveal Boundaries between Species Closely Related to Cryptococcus Pathogens
Source: mBio. 2019 Jun 11;10(3):e00764-19. doi: 10.1128/mBio.00764-19 (PMC6561019; doi:10.1128/mBio.00764-19)

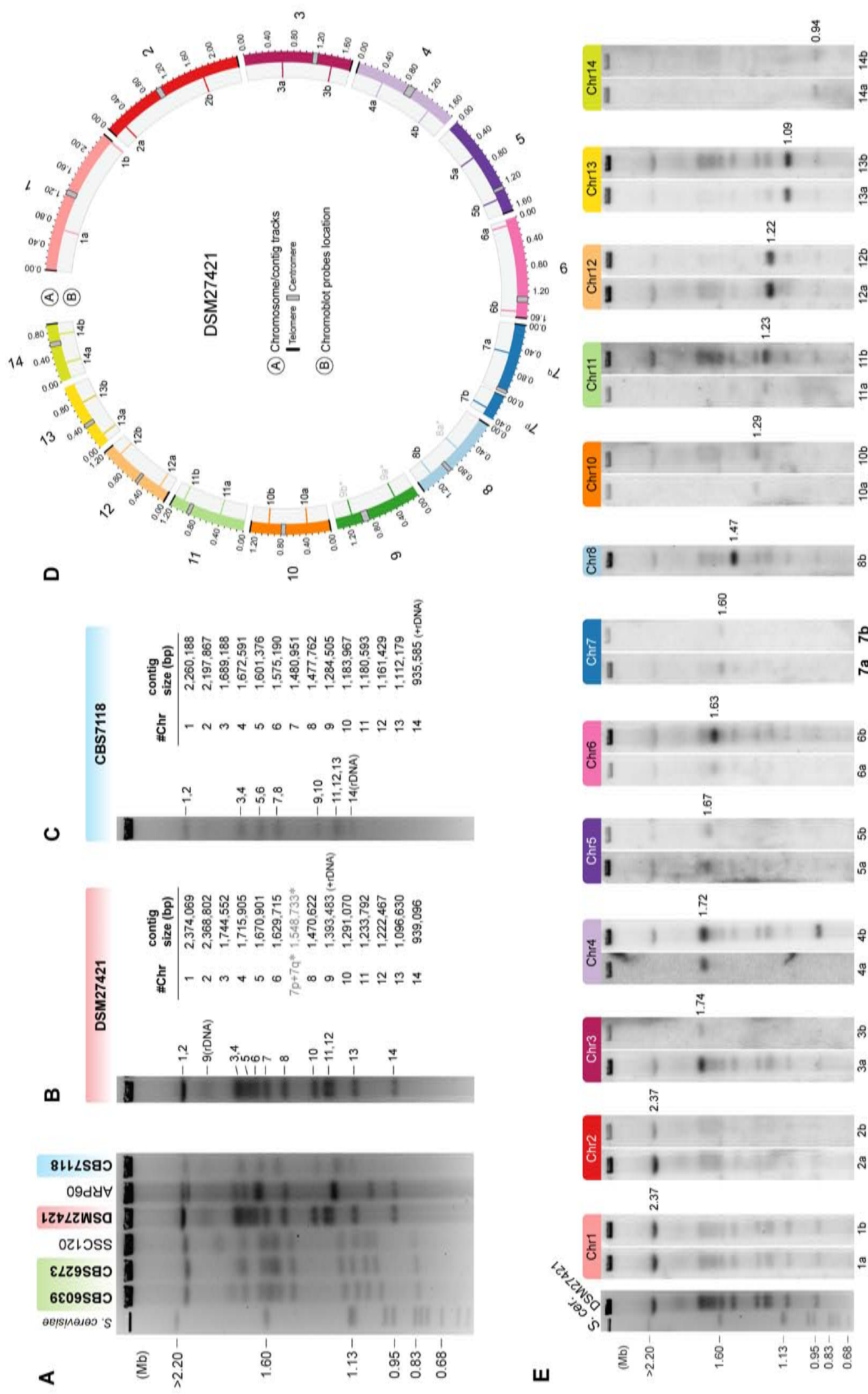

Supplement: FIG S2 [file mBio.00764-19-sf002.pdf]

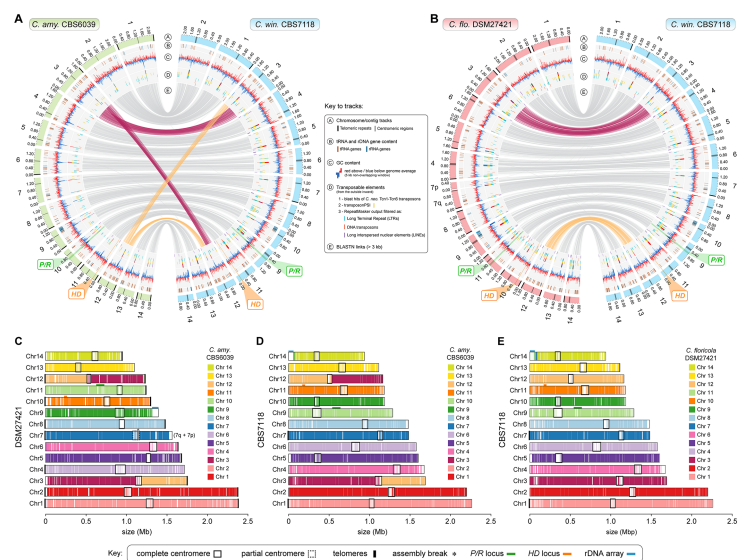

Supplement: FIG S3 [file mBio.00764-19-sf003.pdf]

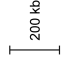

Supplement: FIG S4 [file mBio.00764-19-sf004.pdf]

**A**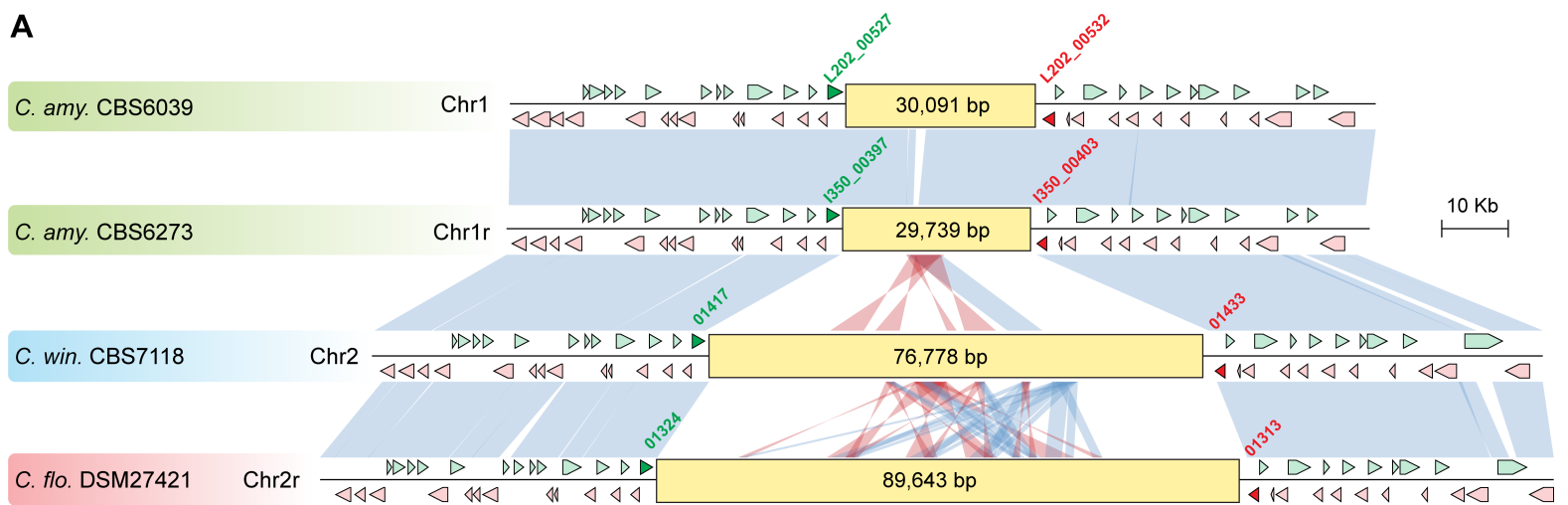**B**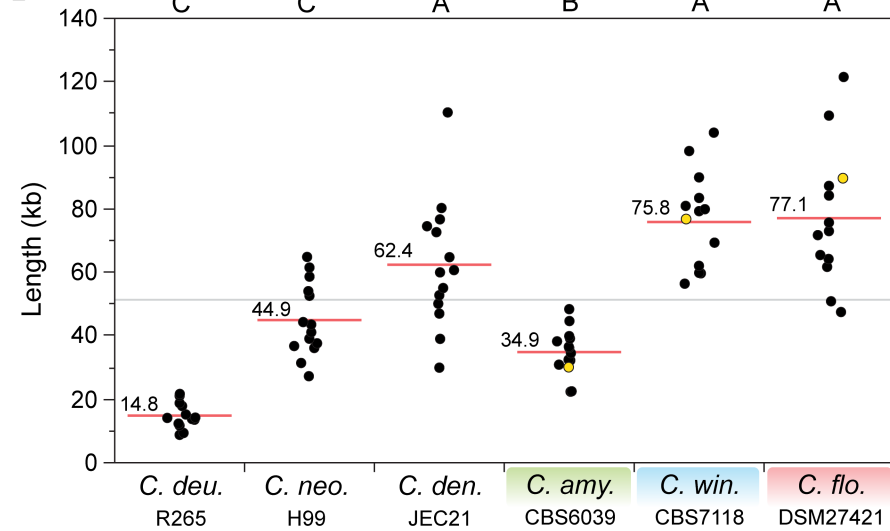

Supplement: FIG S5 [file mBio.00764-19-sf005.pdf]

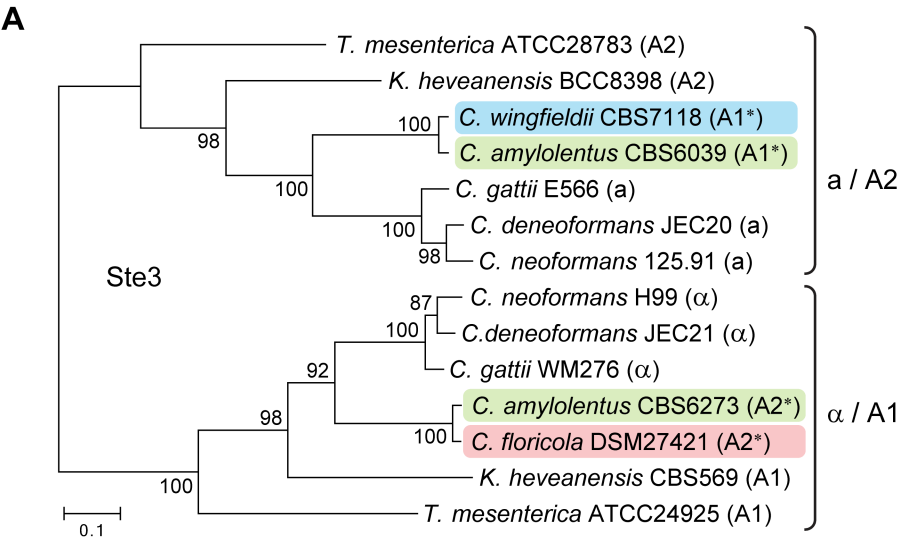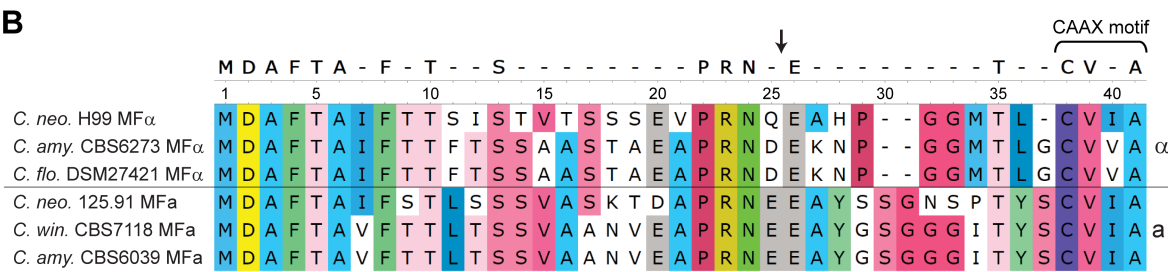

Supplement: FIG S6 [file mBio.00764-19-sf006.pdf]

**A**

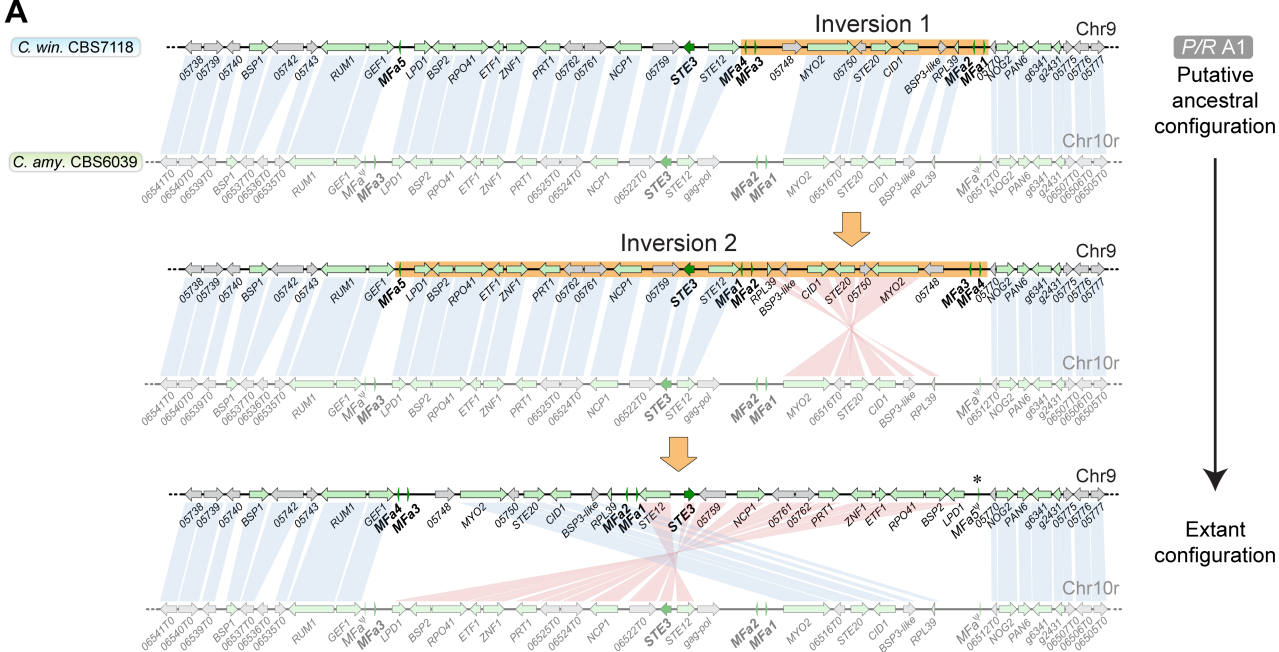

**B**

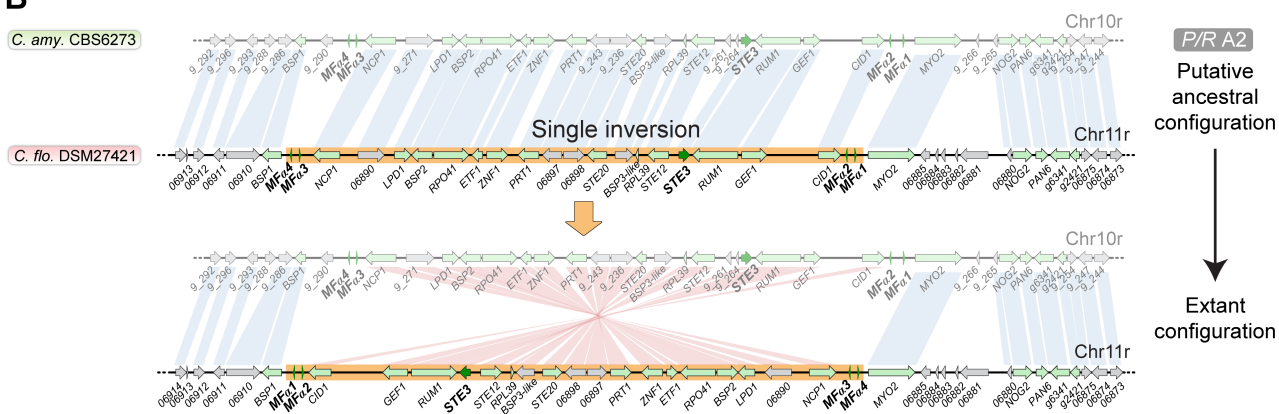

Supplement: FIG S7 [file mBio.00764-19-sf007.pdf]

**A**

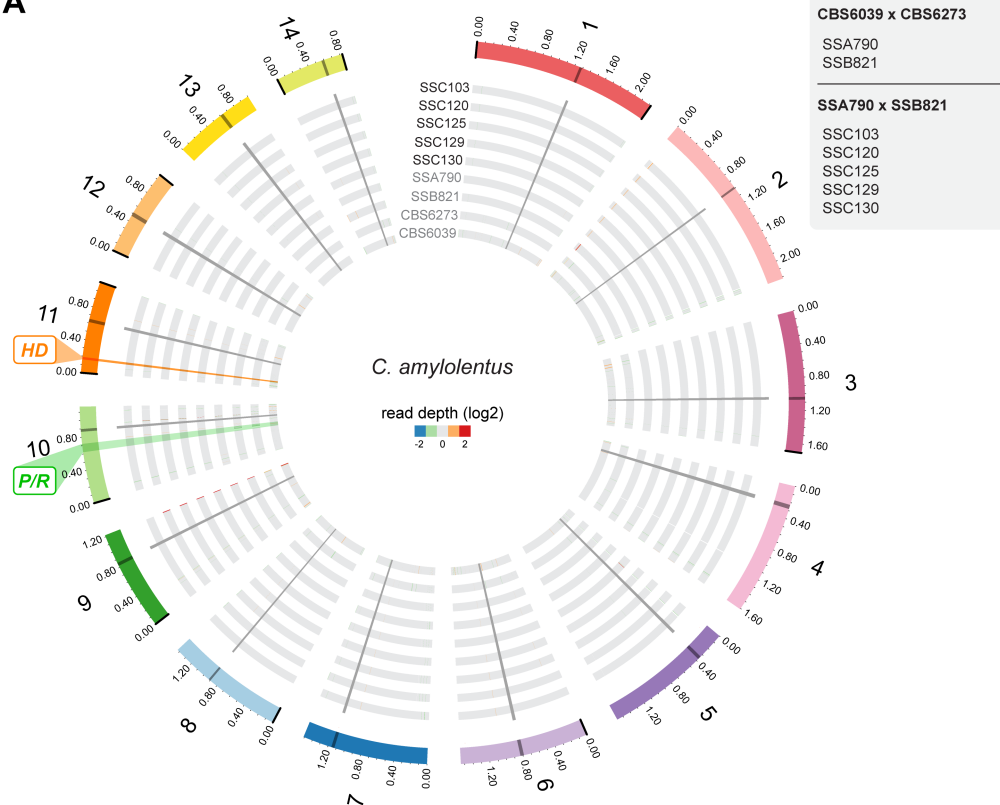

**B**

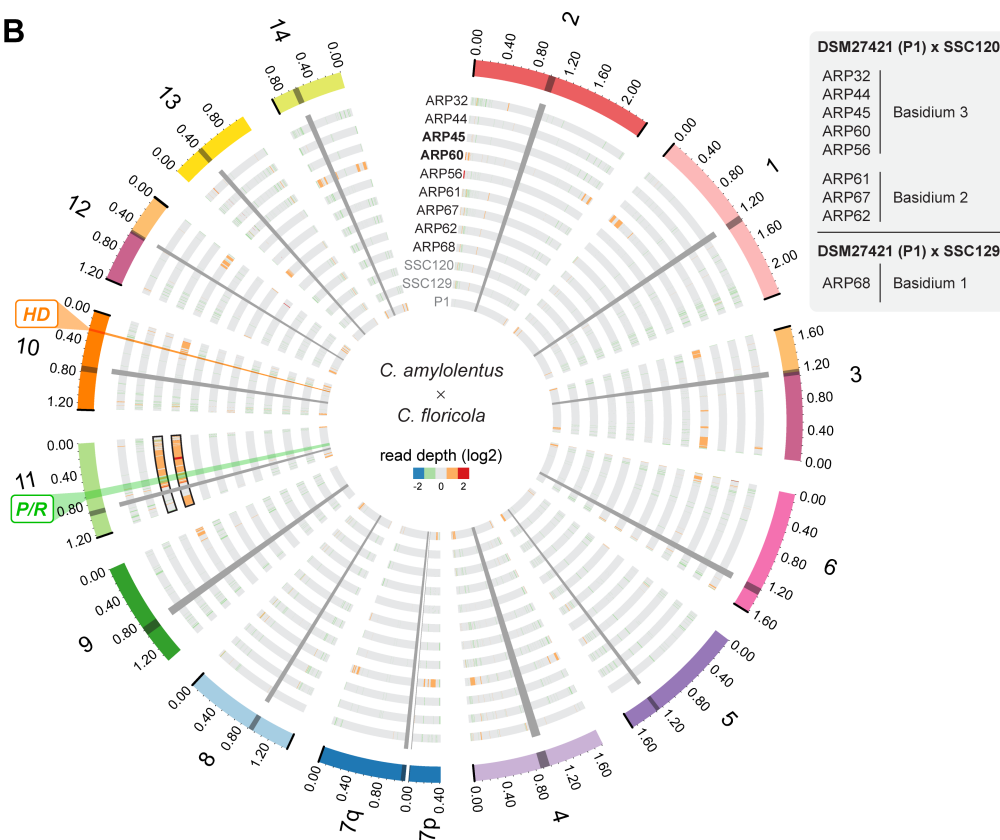

Supplement: FIG S8 [file mBio.00764-19-sf008.pdf]

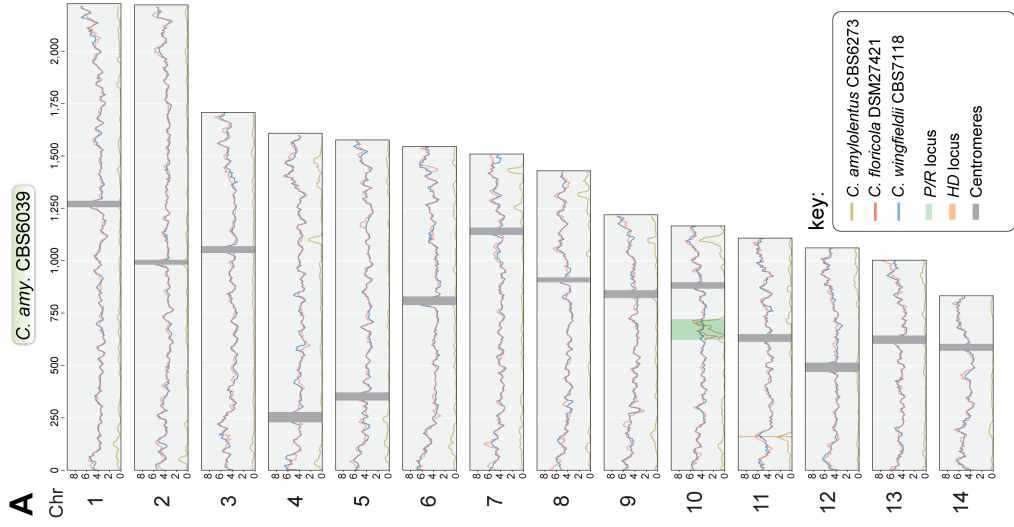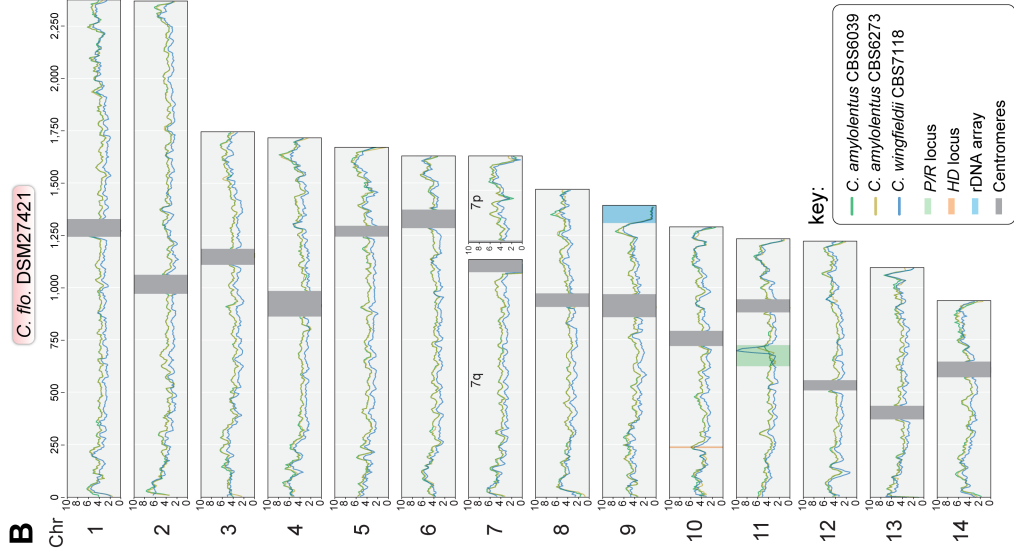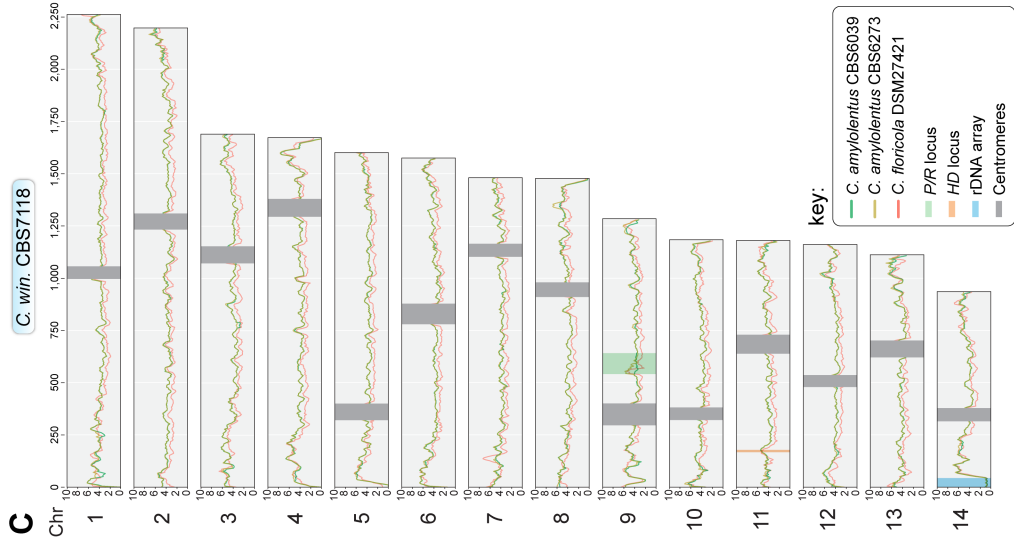

Supplement: FIG S9 [file mBio.00764-19-sf009.pdf]
